# Development of a Novel Combined Wind–Rain Piezoelectric Energy Harvester

**DOI:** 10.3390/s26196271

**Published:** 2026-10-03

**Authors:** Yuansheng Chen, Hao Lou, Yiyang He, Jian Wang, Shaona Liu

**Affiliations:** 1 School of Mechanical Engineering, Yancheng Institute of Technology, Yancheng 224051, China; chenys@njust.edu.cn (Y.C.); 19827066706@163.com (H.L.); 17715500429@163.com (Y.H.); 2 School of Energy and Power Engineering, Nanjing University of Science and Technology, Nanjing 210094, China; wjnjust801@163.com

**Keywords:** piezoelectric energy harvester, hybrid wind–rain energy harvesting, vortex-induced vibration, P-SSHI circuit, finite element analysis

## Abstract

Aiming at the unstable power output of single-source piezoelectric energy harvesters for ecological monitoring in bird nature reserves, this work develops a novel combined wind–rain piezoelectric energy harvester and establishes a complete numerical modeling framework. The proposed device integrates a snowflake-shaped bluff body for broadband vortex-induced wind energy capture and a self-tilting water container for raindrop gravitational potential energy collection, with both excitation sources equipped with independent piezoelectric cantilevers that share a unified passive parallel-synchronized switch harvesting on inductor (P-SSHI) interface circuit for coordinated energy conversion of two ambient energy sources. A full set of closed-loop theoretical formulas covering rain potential energy, vortex-induced aerodynamic excitation, electromechanical coupling, and LC-resonance-based voltage inversion is derived. Static finite element analysis, Multisim circuit simulation, and oscilloscope-based bench-scale experiments are implemented to evaluate structural deformation, stress distribution, output voltage, impedance-matched power, and steady-state load performance. Simulation and experimental results validate the rationality of the structural scheme and mathematical model. All validations are performed under laboratory-simulated conditions, and long-term field meteorological tests are reserved for future investigations. The compact low-noise proof-of-concept prototype shows application potential for field monitoring nodes, and the established modeling framework provides design references for multi-source kinetic-energy harvesters.

## 1. Introduction

In the context of the global energy structure transitioning towards clean and low-carbon energy, wind energy and rainwater can be regarded as widely distributed renewable energy sources with significant development potential [1]. With the rapid development of micro-electromechanical systems, the demand for distributed power supply from miniature wireless sensors and portable electronic devices is increasingly urgent [2]. Traditional large-scale power generation equipment is limited by installation space and scene adaptability, making it difficult to meet the energy supply demands of such miniature electronic devices [3]. In wind power generation technology, the piezoelectric wind energy harvester based on flow-induced vibration has attracted attention due to its compact structure and ease of miniaturization [4]. However, the traditional cylindrical bluff body has problems such as a narrow locking range and a decrease in energy conversion efficiency at high wind speeds [5]. Rainwater collection technology is mainly divided into raindrop impact type and potential energy collection type [6]. The former has limitations such as small single-drop energy and low conversion efficiency, while the latter can enhance energy density through water storage release but lacks a collaborative utilization scheme with other energy forms [7]. Current research mostly focuses on the optimization of single-energy capture, such as using a snowflake-shaped bluff body to increase the locking range and power density of wind energy capture or strengthening potential energy capture through self-tilting water tanks [8]. However, no integrated power generation device that combines wind and rain energy for coordinated operation has been designed [9]. Nevertheless, few existing works have developed matching interface circuits that can simultaneously handle mixed wide-frequency and pulse-type outputs generated by both wind- and rain-induced excitations. Based on this, this work integrates the collection advantages of the two types of energy and designs a composite power generation device based on wind and rain energy [10]. Through structural integration and collaborative control, it achieves efficient capture and complementary utilization of the two renewable energies with the potential to provide stable distributed power supply for miniature electronic sensors [11]. It is particularly suitable for ecologically sensitive areas such as bird nature reserves without interfering with bird habitats or damaging the original environment, providing clean and noise-free power supply for field monitoring equipment [12] and effectively expanding the application scenarios and practical value of renewable energy [13].

Therefore, the development of hybrid energy harvesters capable of capturing multiple ambient energy sources has become a research hotspot. This work integrates two energy-harvesting techniques for raindrop kinetic energy and wind energy, enabling continuous operation under diverse weather conditions. Compared with single-source harvesters, the multi-source hybrid device greatly boosts the stability and reliability of output power, lessens dependence on external power supplies and batteries, and cuts down carbon emissions together with environmental contamination. The proposed integrated harvester features simple raw-material requirements and fabrication workflows for mass production, outstanding environmental merits and promising sustainable development prospects. For ecological-monitoring applications within bird nature reserves, it is expected to deliver all-weather, maintenance-free power for environmental sensors, wildlife-behavior monitoring terminals and wetland-water-quality testing gear. This setup addresses the wiring obstacles and cumbersome battery replacement work in secluded eco-sensitive zones and substantially expands the application scope of Internet of Things sensing terminals.

The development of this combined raindrop and wind power generation device has clear practical significance. On the one hand, it can efficiently utilize natural energy, alleviate the power supply problems of outdoor ecological-monitoring equipment, provide independent and reliable power solutions for various low-power sensing devices in the core area, buffer zone, and monitoring corridor of bird protection areas, and reduce reliance on traditional power grids and disposable batteries. On the other hand, the device’s feature of having no rotating parts and silent operation can minimize interference with bird activities, promote the application of eco-friendly energy technologies, contribute to the sustainable development of protected areas, and reduce greenhouse gas emissions and environmental pollution. Integrating the two power generation methods not only optimizes energy collection efficiency but also builds a distributed power supply scheme adaptable to multiple scenarios, providing key technical support for green and low-interference ecological-monitoring networks.

### 1.1. Research Progress of Raindrop Energy Harvester

**Figure 1:**
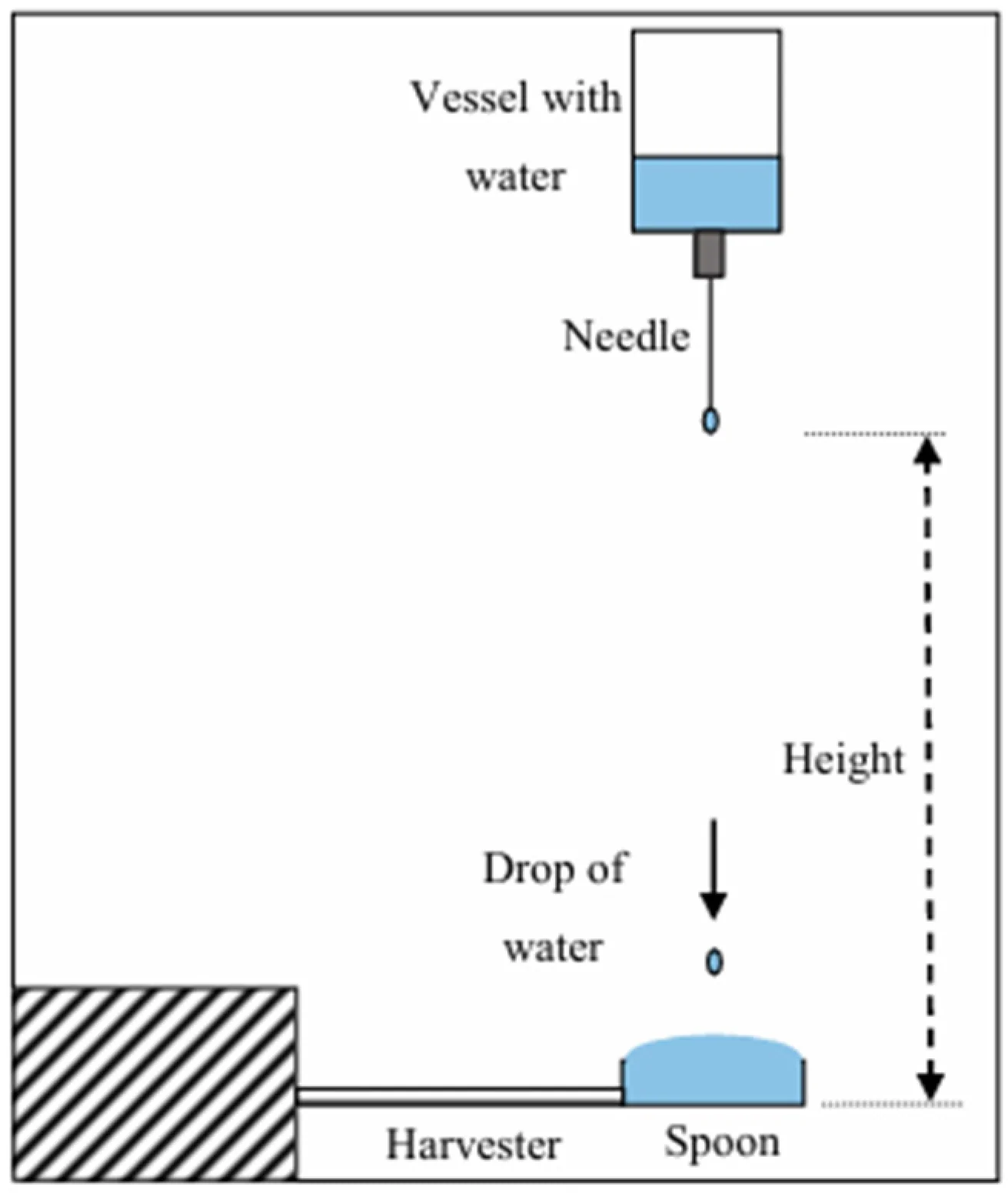
Structure and working principle of the raindrop energy harvester. Reproduced from Ref. [1], licensed under the Creative Commons Attribution (CC BY 4.0) license.

Currently, various countries have achieved remarkable results in the field of rainwater power generation. Alberto Doria et al. designed a novel piezoelectric raindrop energy harvester that is unique [1]. This harvester combines a cantilever beam with a rectangular spoon filled with water. When a raindrop strikes the water surface inside the spoon, it triggers a series of vibration responses, thereby achieving energy collection [14]. The structural layout and working mechanism of this traditional spoon-type raindrop harvester are intuitively displayed in Figure 1. From a theoretical perspective, when a raindrop impacts a solid surface, much of the energy is lost due to phenomena such as splashing, while when it impacts a liquid surface, the energy transfer ratio significantly increases. Experimental measurements have shown that when a spoon filled with water is struck by raindrops, the average open-circuit voltage reaches 3.22 V, which is much higher than the 1.03 V of a dry spoon, fully demonstrating the advantages of liquid surface impact. Moreover, when the optimal load resistor is connected, the collected energy is higher than that of most traditional simple harvesters [15]. This design effectively optimizes the energy collection process and provides a new idea for raindrop power generation technology [16]. However, this structure can only generate power under rainfall conditions. Without rain, it cannot produce electricity, and it has not been combined with other energy forms, making it difficult to meet the all-day stable power supply demands of outdoor equipment [17].

### 1.2. Research Progress of Wind Vibration Energy Harvester

**Figure 2:**
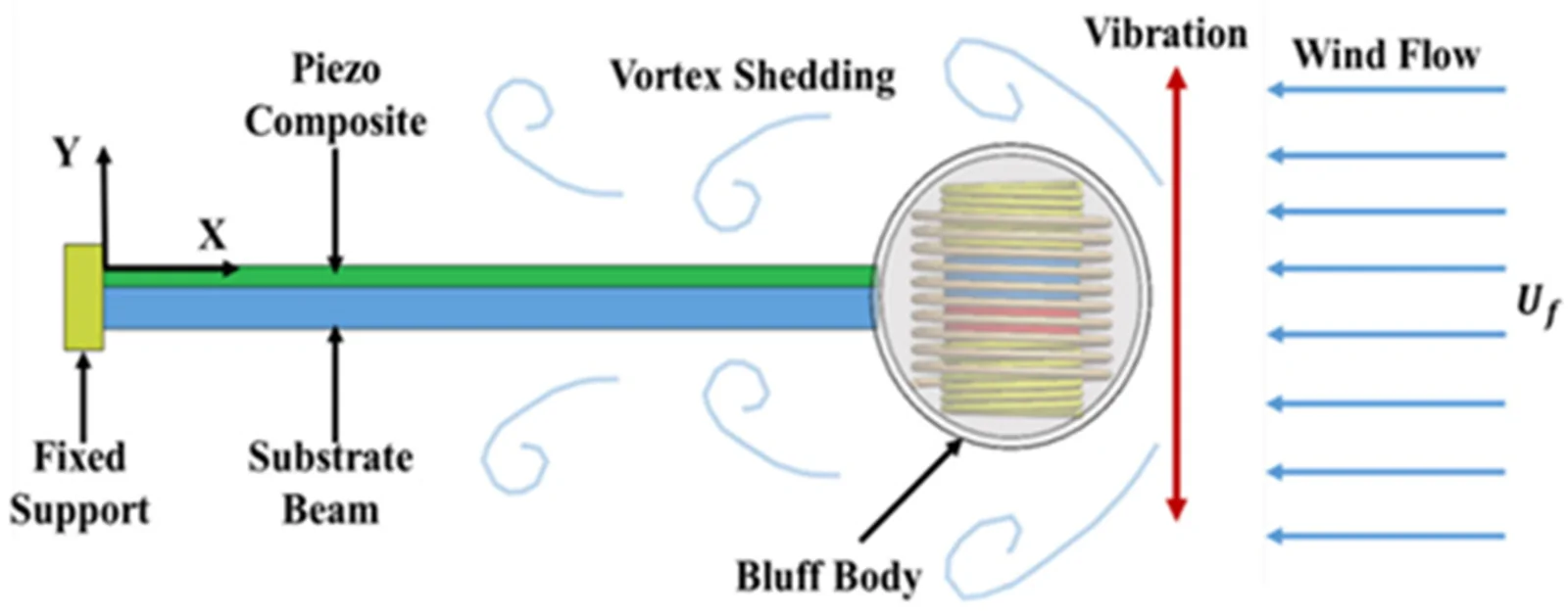
Structure and working principle of the wind energy harvester. Reproduced from Ref. [2], licensed under the Creative Commons Attribution (CC BY 4.0) license.

Scholars worldwide are constantly exploring and innovating in the field of wind power generation. AlRiyami Mahmood et al. proposed a piezoelectric and electromagnetic hybrid wind energy harvester, which utilizes the internal space of the bluff body and integrates multiple piezoelectric and electromagnetic energy capture units [2]. The overall structural layout and vortex-induced vibration working principle of this traditional wind harvester are illustrated in Figure 2. A comprehensive coupled dynamic model was established using Lagrange’s formula to deeply study the influence of various parameters on harvester performance [18]. The research found that reducing the mass of the composite beam can significantly increase maximum output power and bandwidth. The electromagnetic coupling coefficient plays a significant role in improving the bandwidth, and under certain conditions, it can greatly boost captured power at high wind speeds. However, this structure is complex and difficult to integrate. It also fails to consider adaptability to rainy environments, so coordinated power supply under mixed wind and rain conditions cannot be realized, and it lacks long-term field stability.

To solve these problems, this paper designs an integrated wind and rain dual-source piezoelectric energy harvester. This device overcomes the disadvantages of traditional single wind or rain harvesters such as poor environmental adaptability, cumbersome installation and large volume as well as obvious vibration interference. It is developed to adapt to the complex and harsh wild working conditions of bird nature reserves and possesses prominent engineering innovation value and broad application prospects. Through three-dimensional integrated box modeling, the funnel, self-tilting container and piezoelectric cantilever beam are highly integrated inside the sealed housing [19]. This design greatly simplifies field installation and improves energy collection efficiency via the top radial drainage structure. The compact structure has no sharp exposed components, which avoids disturbing bird resting activities and damaging the original ecological environment of the reserve. The hexagonal snowflake-shaped bluff body is mounted externally and closely connected with the piezoelectric cantilever beam [20]. The two energy capture units operate without mutual interference, and optimized windward angles strengthen vortex-induced vibration to expand the effective wind energy capture range for variable wind conditions in reserves. Aiming at the obvious mismatch of output characteristics between wind and rain energy, an adaptive three-stage energy management circuit is adopted to solve the compatibility problem of traditional circuits with high-voltage pulses and wide-frequency alternating signals. Combined with the P-SSHI energy recovery circuit and supercapacitor energy storage, it realizes efficient rectification, voltage stabilization and coordinated stable output of dual energy sources. This harvester puts forward a new design idea for miniature composite energy capture devices and can provide stable power support for IoT monitoring terminals and ecological wireless sensing equipment. It has important practical significance for promoting large-scale application of distributed clean energy in bird ecological field monitoring and comprehensive ecological perception.

## 2. Operating Principle and Structural Design

### 2.1. Operating Principle

When raindrops fall and hit the baffle, a series of physical phenomena such as water surface fluctuation and splashing occur [1]. These phenomena trigger vibration and mechanical deformation of the piezoelectric cantilever beam [14]. According to the piezoelectric effect, the piezoelectric cantilever beam generates electric charges during deformation to convert raindrop kinetic energy into electric energy [19]. Rainwater flows into the self-tilting container through the top radial funnel. As the water volume rises, the container’s gravity center shifts to trigger automatic overturning [20]. The discharged water flow impacts the piezoelectric cantilever baffle to induce beam vibration. Piezoelectric patches convert mechanical strain into electric energy via the piezoelectric effect, and the generated electricity is rectified and stabilized by the control and storage module before being stored in supercapacitors. After water discharge, the container resets under gravity to start a new energy collection cycle. The generated electric energy passes through the energy management and conversion circuit for rectification and filtering, and then is stored in rechargeable batteries or supercapacitors to supply power for sensor terminals.

**Figure 3:**
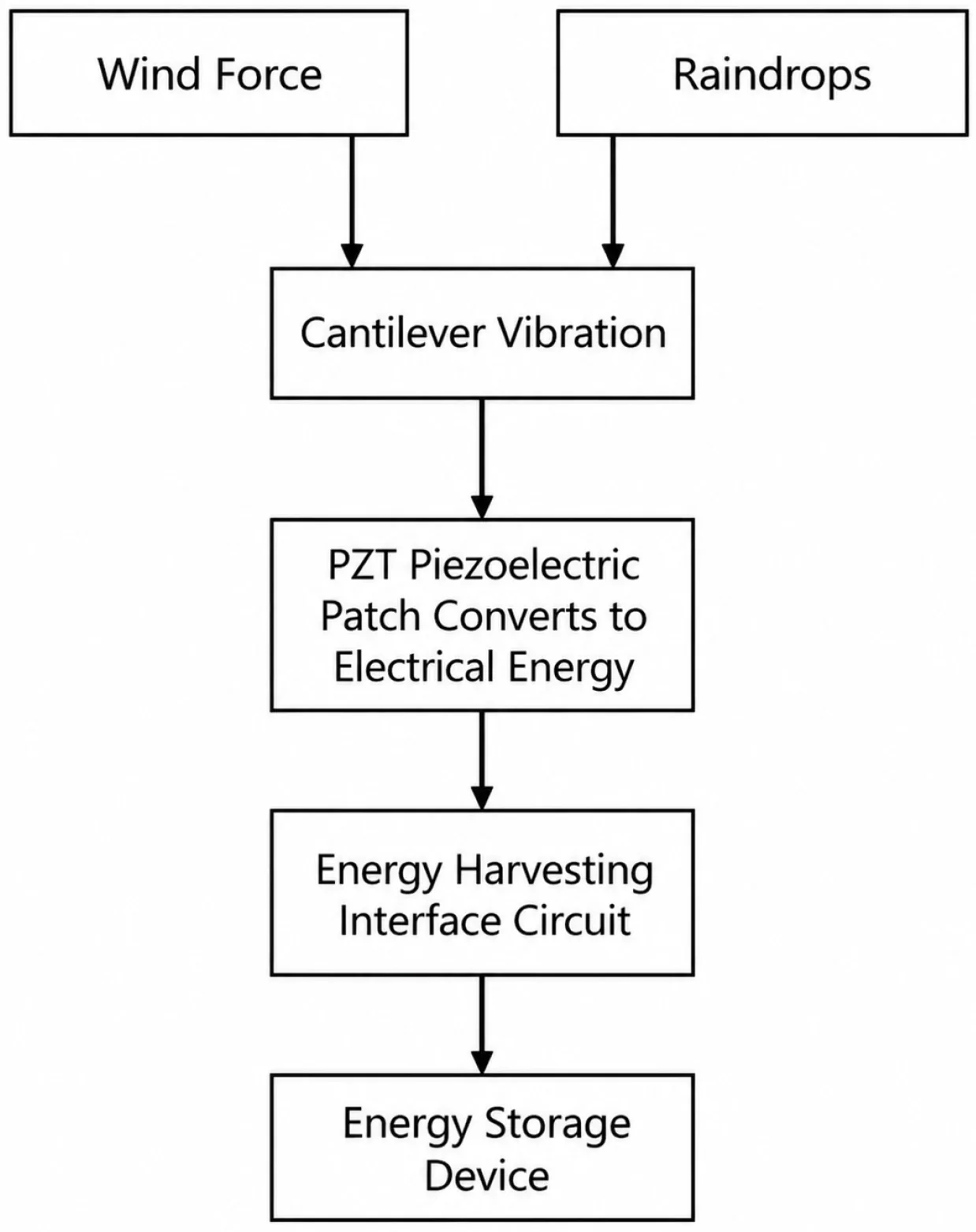
Operating principle diagram.

When wind flows through the bluff body structure, alternating vortices form at the leeward side [2]. The vortex shedding effect induces bluff body vibration [18]. Airflow acts on the hexagonal snowflake-shaped bluff body to generate vortex-induced vibration. Vortex shedding on the bluff body surface drives piezoelectric patches to produce strain electricity. Electric energy generated from wind and rain excitation is processed by the same energy management circuit to realize coordinated output. The complete energy conversion flow under dual wind and rain excitation is visualized in Figure 3.

### 2.2. Structural Design

**Figure 4:**
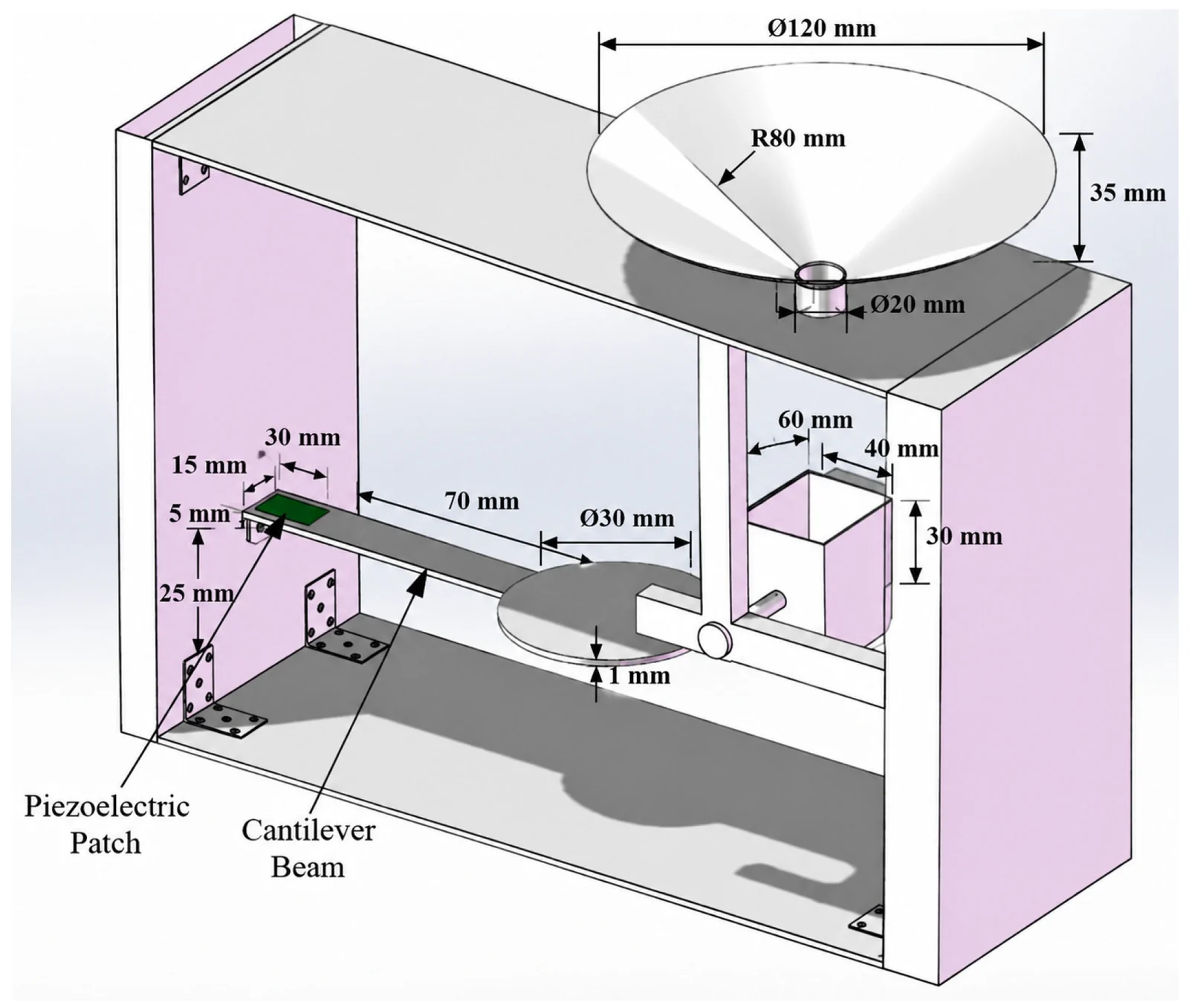
Structure of rain energy harvesting module.

The raindrop energy capture module is arranged at the bottom of the whole device and equipped with a large-diameter radial funnel. Three-dimensional geometric modeling optimizes the funnel surface curvature to realize rapid rainwater drainage; specifically, the funnel features a top inlet diameter of 120 mm, a bottom outlet diameter of 20 mm, an overall axial height of 35 mm, and a curved diversion surface with a radius of 80 mm to ensure efficient raindrop convergence and drainage. A self-tilting water storage container is installed under the funnel. Its structural dimensions are optimized through simulation, with an effective internal cuboid cavity of 60 mm (length) × 40 mm (width) × 30 mm (height), corresponding to an effective water storage volume of 72 mL. The rotation shaft is arranged on the side wall at a height of 12 mm from the bottom, and an 8 g counterweight block with a 25 mm moment arm is fixed on the opposite side of the shaft to adjust the gravity center for automatic tipping and water release. Piezoelectric cantilever beams are placed below the container, with a vertical distance of 25 mm between the cantilever fixed end and the container water outlet. The cantilever substrate is made of brass with dimensions of 70 mm × 20 mm × 0.3 mm (length × width × thickness), and a circular baffle (30 mm in diameter, 1 mm in thickness) is fixed at the beam end, coaxially aligned with the upper water outlet, to bear water flow impact and realize mechanical to electric energy conversion. One PZT-5H piezoelectric patch (30 mm × 15 mm × 0.2 mm) is bonded on the upper surface of the beam near the fixed end, with the bonding edge 5 mm away from the clamping position. Its structural layout is shown in Figure 4, where the cantilever beam with piezoelectric material is clearly marked.

**Figure 5:**
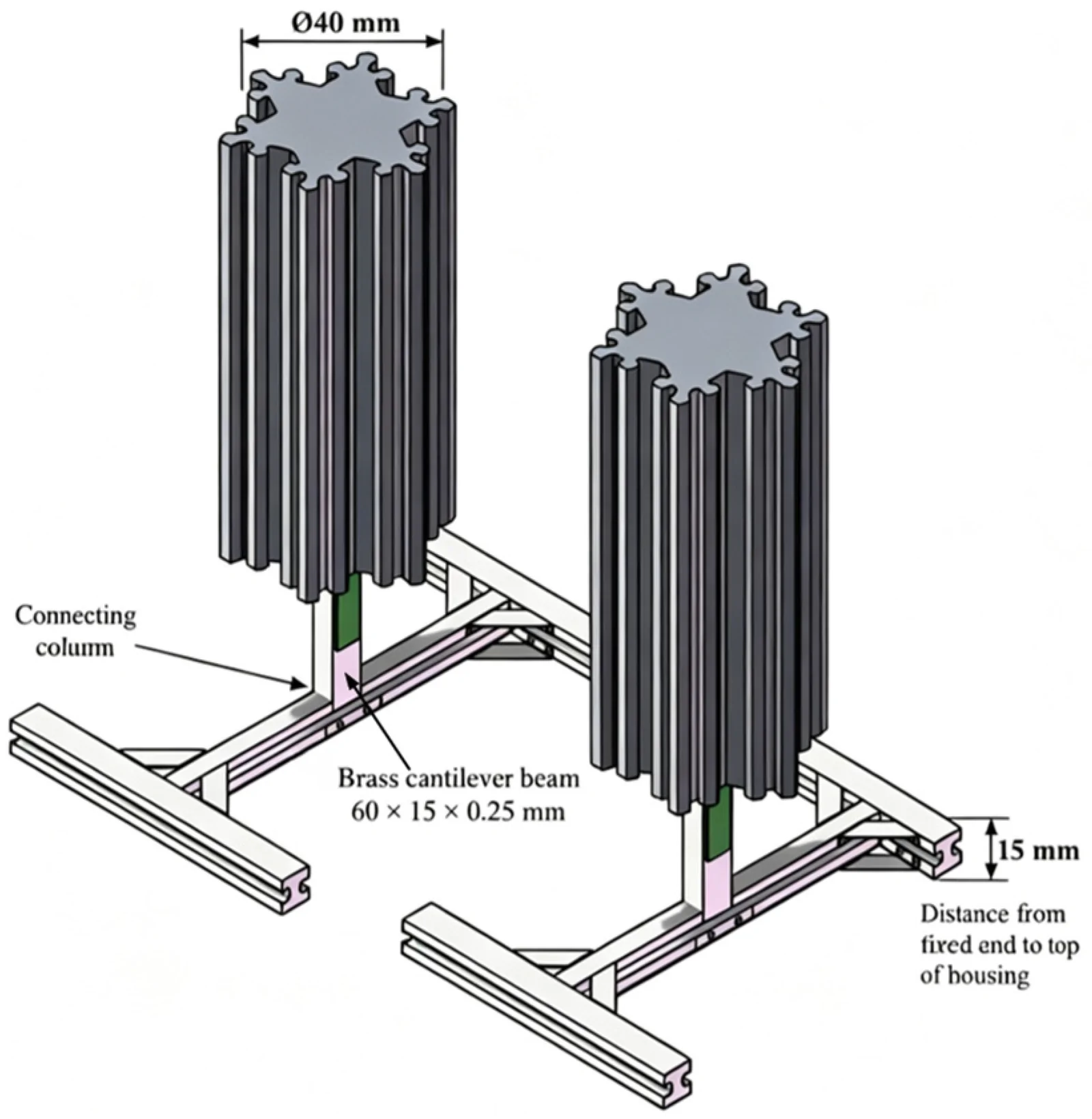
Structure of wind energy harvesting module.

The wind energy capture module is mounted on the upper section of the device housing. Its core component is a hexagonal snowflake-shaped bluff body oriented toward the incoming wind. The regular hexagonal main body has a circumscribed circle diameter of 40 mm, with six branches each extending 15 mm outwards at a thickness of 2 mm. The bluff body is connected to the free end of the wind-induced cantilever via a 10 mm long connecting column. This module shares a supporting frame with the rain energy harvesting unit. The wind-induced cantilever uses a brass substrate with dimensions of 60 mm × 15 mm × 0.25 mm (length × width × thickness), and the vertical distance between its fixed end and the top surface of the housing is 15 mm. The snowflake-shaped bluff body is 3D-printed from PA12 engineering plastic, with geometric dimensions and material properties fully consistent with the finite element simulation model in Chapter 3. This compact layout avoids mutual vibration interference between the two energy harvesting modules, and its structure is illustrated in Figure 5.

**Figure 6:**
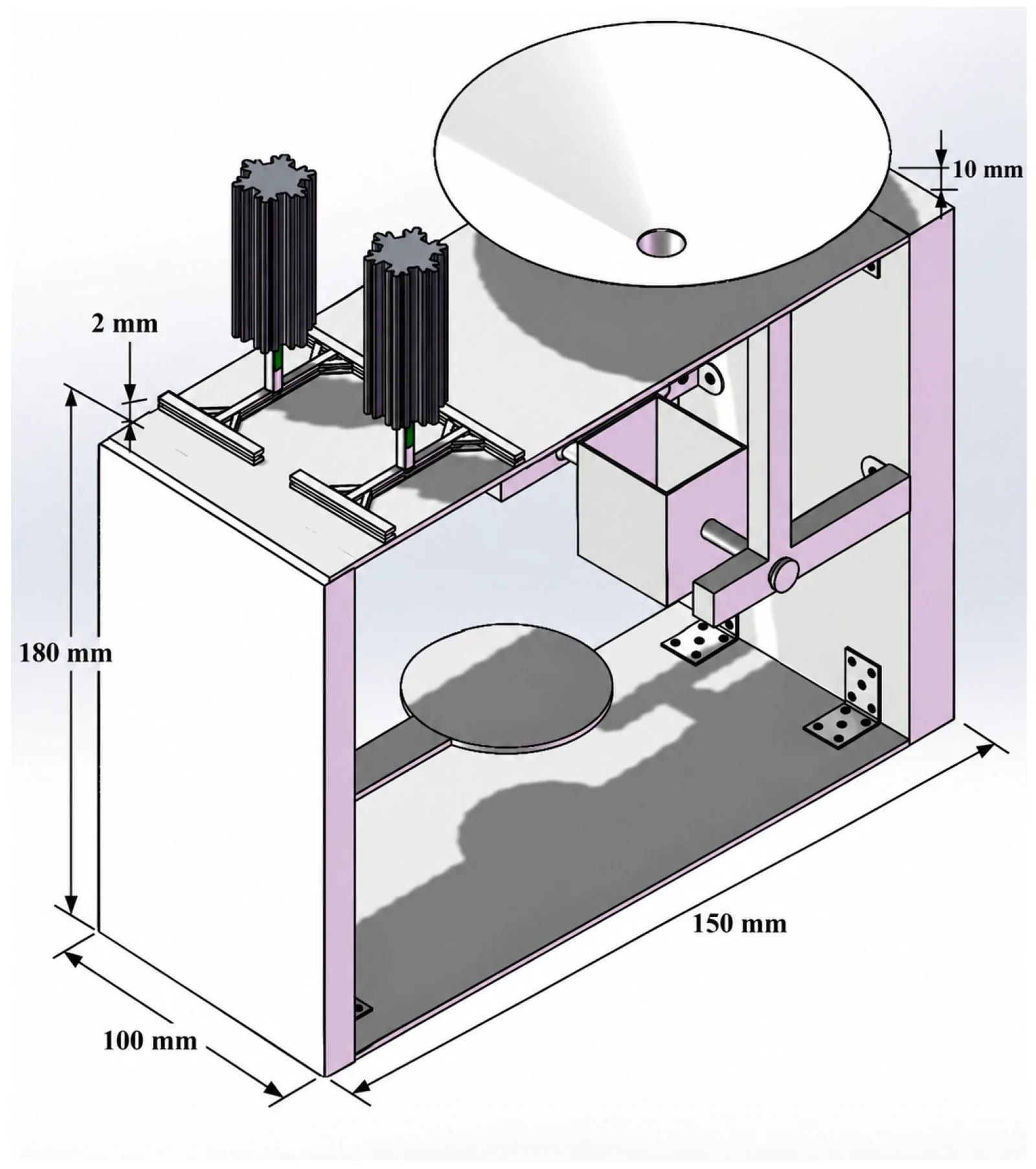
Integrated hybrid wind–rain piezoelectric energy harvesting prototype.

An integrated sealed box structure is adopted with an acrylic main frame. The top funnel, upper wind energy capture unit and intermediate rain energy conversion unit are seamlessly connected inside the housing. The overall dimensions of the integrated prototype are 150 mm (length) × 100 mm (width) × 180 mm (height), with a shell wall thickness of 2 mm, and the top funnel protrudes 10 mm above the upper surface of the shell. The overall size and weight are moderate for convenient field deployment, as shown in Figure 6.

## 3. Finite Element Analysis of Core Energy-Harvesting Components

This chapter presents static structural finite element analyses of the core energy-harvesting components using ANSYS Workbench, with consistent modeling configurations adopted for both the wind-induced bluff body model and the rain-impact cantilever model. For the wind excitation model, the aerodynamic load acting on the bluff body is simplified as an equivalent static lift force derived from the vortex-induced aerodynamic formula. The average lift force, with a total resultant value of 0.11 N, is applied as a uniform surface load over the windward face of the bluff body. For the rain impact model, the water flow impact is equivalent to a static concentrated force, which is loaded at the center of the circular receiving plate at the free end of the cantilever beam. The clamped end of each cantilever substrate is defined as a fixed support constraint that restricts all translational and rotational degrees of freedom. All mating interfaces between the bluff body and connecting column, the connecting column and cantilever substrate, and the piezoelectric patch and substrate are set as bonded contact, without consideration of interfacial slip or delamination. Specifically, the bluff body and connecting column are made of PA12 engineering plastic, the cantilever substrate uses brass, and the piezoelectric layer is PZT-5H, with identical settings of elastic modulus, density and Poisson’s ratio. The structural assembly achieves coordinated deformation transfer through the bonded contact settings, and the piezoelectric patch is meshed with piezoelectric coupling elements to realize the electromechanical conversion between mechanical strain and electric field. All material configurations in the simulation are identical to those of the 3D-printed prototype, and no alloy material is used for the bluff body model.

### 3.1. Finite Element Analysis of Wind-Induced Energy-Harvesting Bluff Body

**Figure 7:**
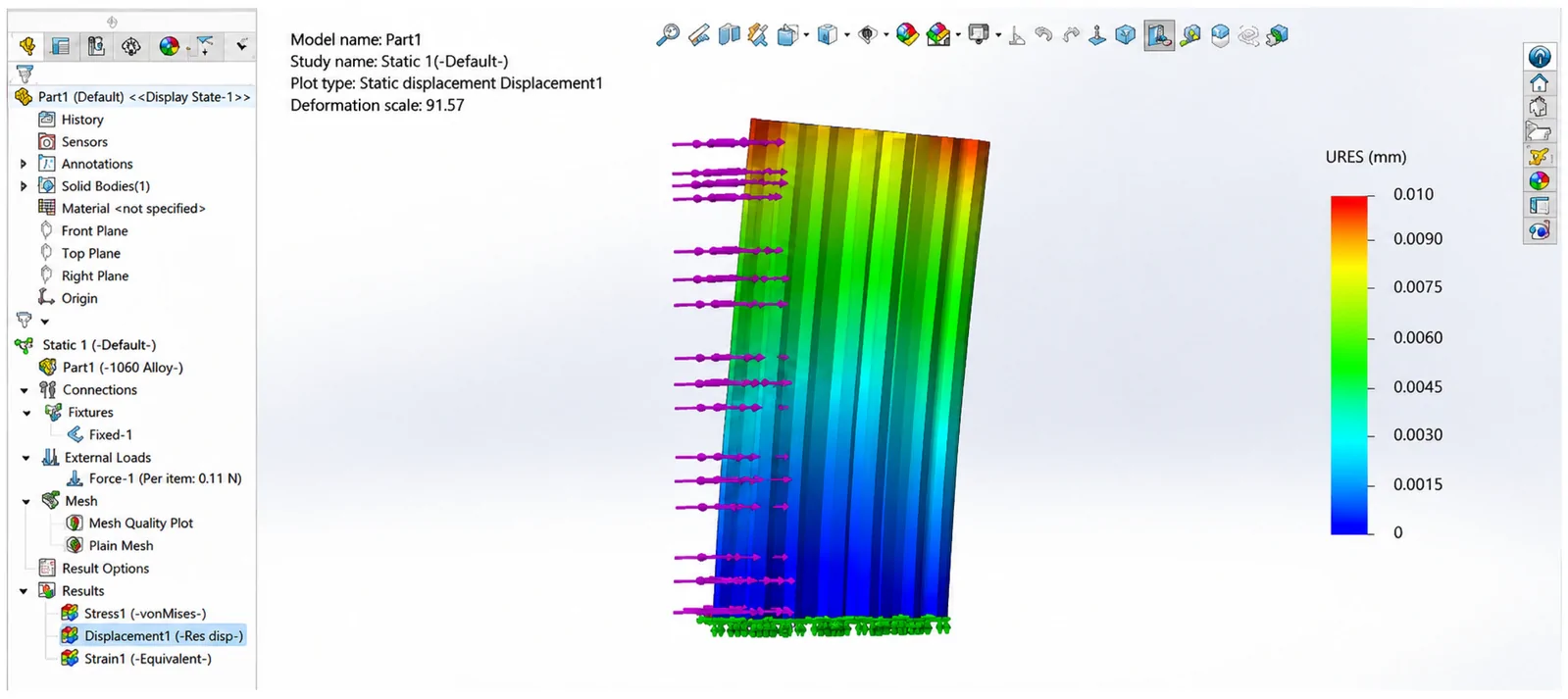
Total-deformation nephogram of snowflake-shaped bluff body under wind excitation.

**Figure 8:**
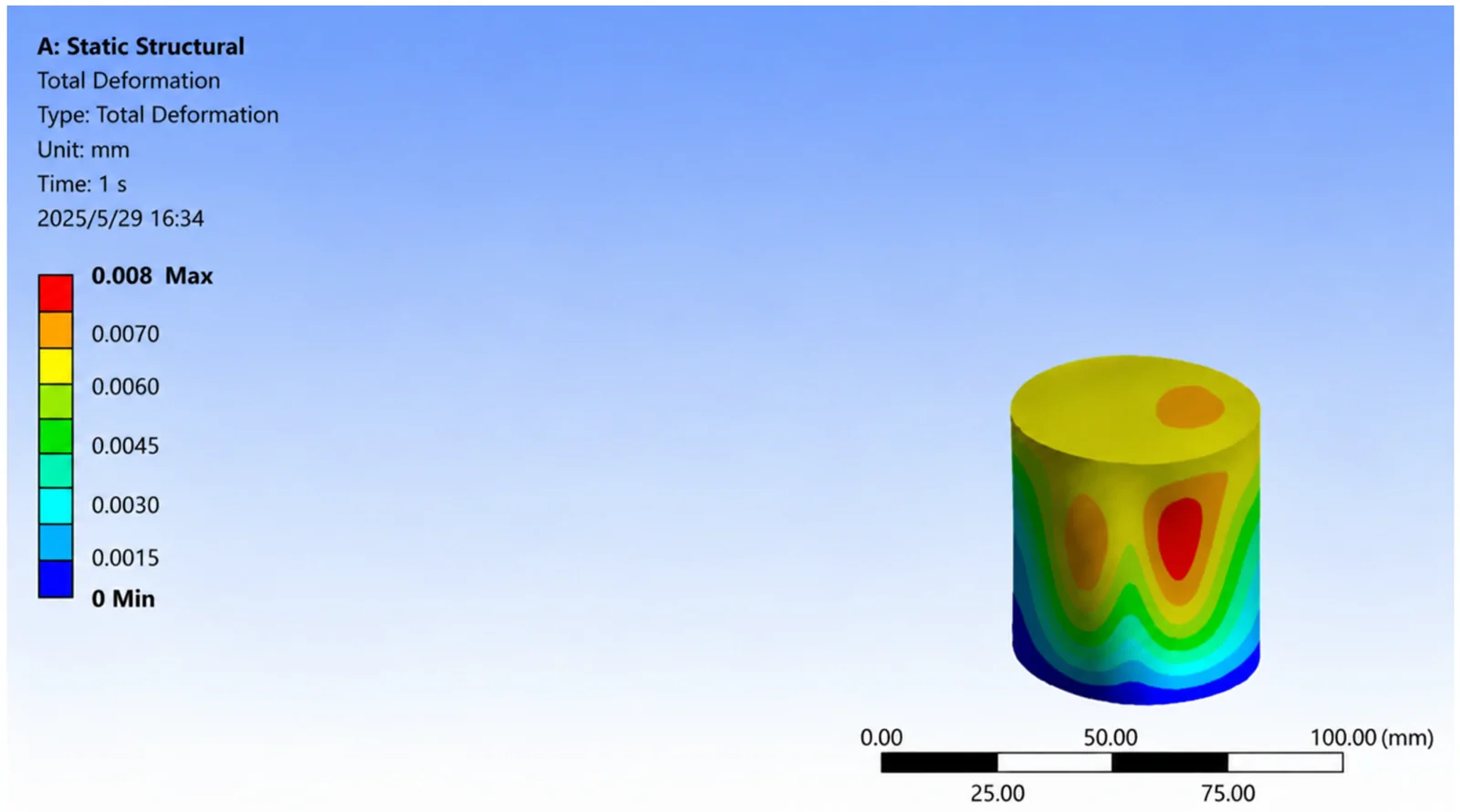
Total-deformation nephogram of cylindrical bluff body under wind excitation.

This section conducts static structural finite element analysis to compare the deformation and strain distribution characteristics of bluff bodies with different configurations under equivalent static wind loads. The analysis aims to provide a structural mechanics basis for bluff body configuration selection, rather than directly evaluating the final vortex-induced vibration energy harvesting performance. Structural deformation results are presented in Figure 7 and Figure 8 and Table 1 and Table 2. Comparative analysis of simulation data shows that the snowflake-shaped bluff body exhibits more favorable structural deformation and strain distribution characteristics compared with the cylindrical counterpart. Under the same equivalent static wind load, the multi-branch snowflake structure produces larger overall deformation with evenly distributed high-strain zones without concentration of local stress. These structural characteristics are beneficial for improving the electromechanical conversion efficiency of piezoelectric materials under vortex-induced vibration and provide a structural basis for superior wind energy harvesting performance. The cylindrical bluff body only generates effective strain in limited local regions, and most areas cannot participate in electromechanical conversion. Meanwhile, local stress concentration increases the risk of structural fatigue under long-term vortex vibration. The multi-branch layout of the snowflake bluff body realizes synchronous vortex shedding on each branch. It features lower structural stiffness, higher vibration sensitivity, larger effective sticking area for piezoelectric patches and better fatigue resistance. Therefore, the snowflake-shaped bluff body is more suitable for wind-induced vibration energy capture. The more uniform strain distribution contributes to stable and efficient piezoelectric electromechanical conversion and improves overall output performance.

### 3.2. Finite Element Analysis of Rain-Impact Piezoelectric Cantilever

This section conducts transient structural finite element analysis to compare the deformation, strain and dynamic response characteristics of two cantilever configurations under time-dependent rain-impact pulse load. Unlike static analysis, transient simulation considers inertial effect and structural damping and can capture the dynamic peak deformation and damped free-vibration behavior after water flow impact. The simulation aims to reveal the dynamic mechanical response under rain-drop impact excitation and provide mechanical basis for cantilever configuration selection for rain energy harvesting.

The pulse impact load corresponding to the water-dumping from the self-tilting container is applied on the center of the circular receiving plate. A fixed support constraint is imposed at the cantilever clamped end. Bonded contacts are defined between the piezoelectric patch and the brass substrate. Transient simulation solves the time-history response within 0–40 ms, and a structural damping ratio ζ = 0.03 is set to reproduce realistic, damped vibration.

**Figure 9:**
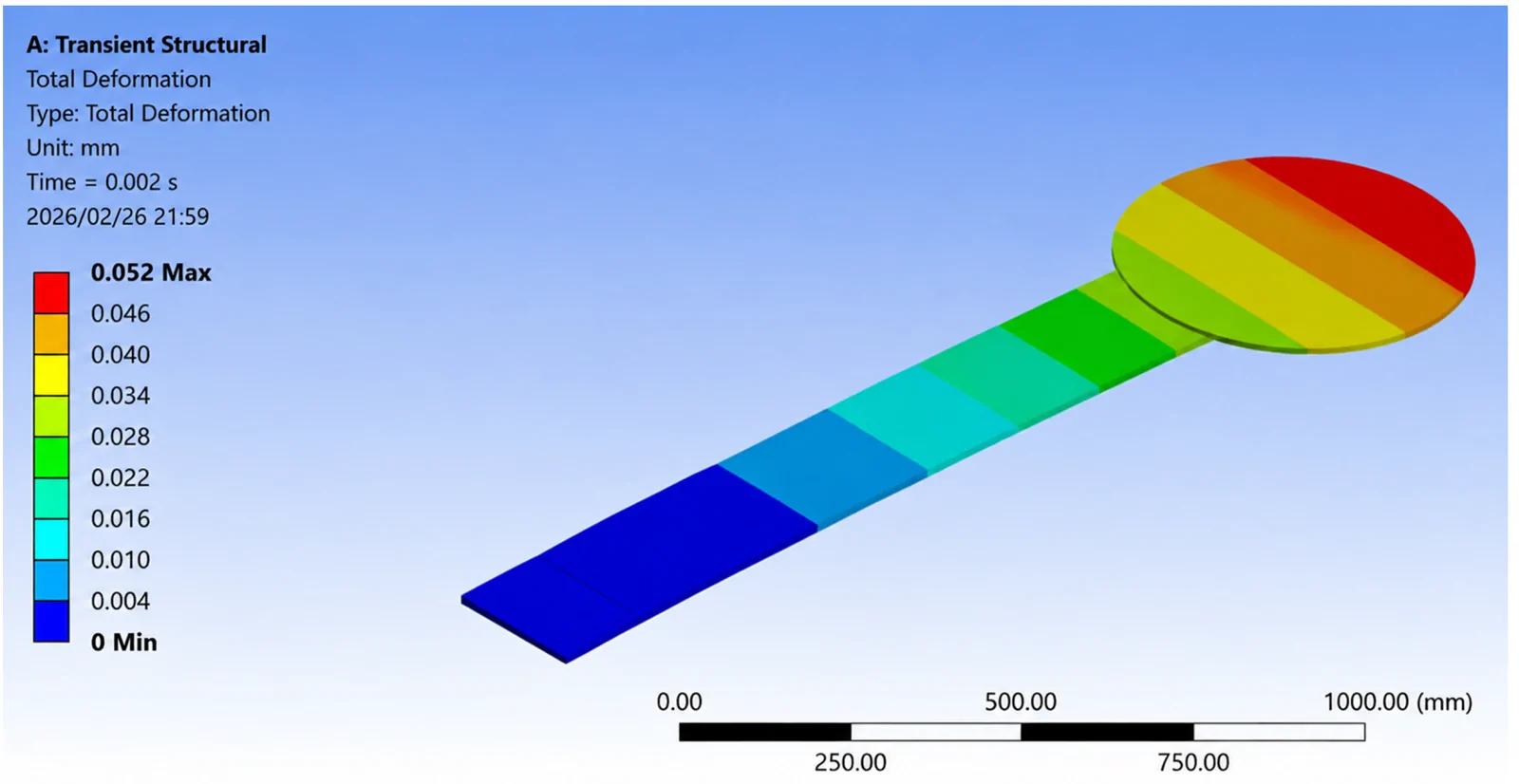
Total-deformation nephogram of optimized cantilever with circular water-receiving plate under rain-impact pulse load, captured at peak-impact instant t = 0.002 s from transient structural simulation.

**Figure 10:**
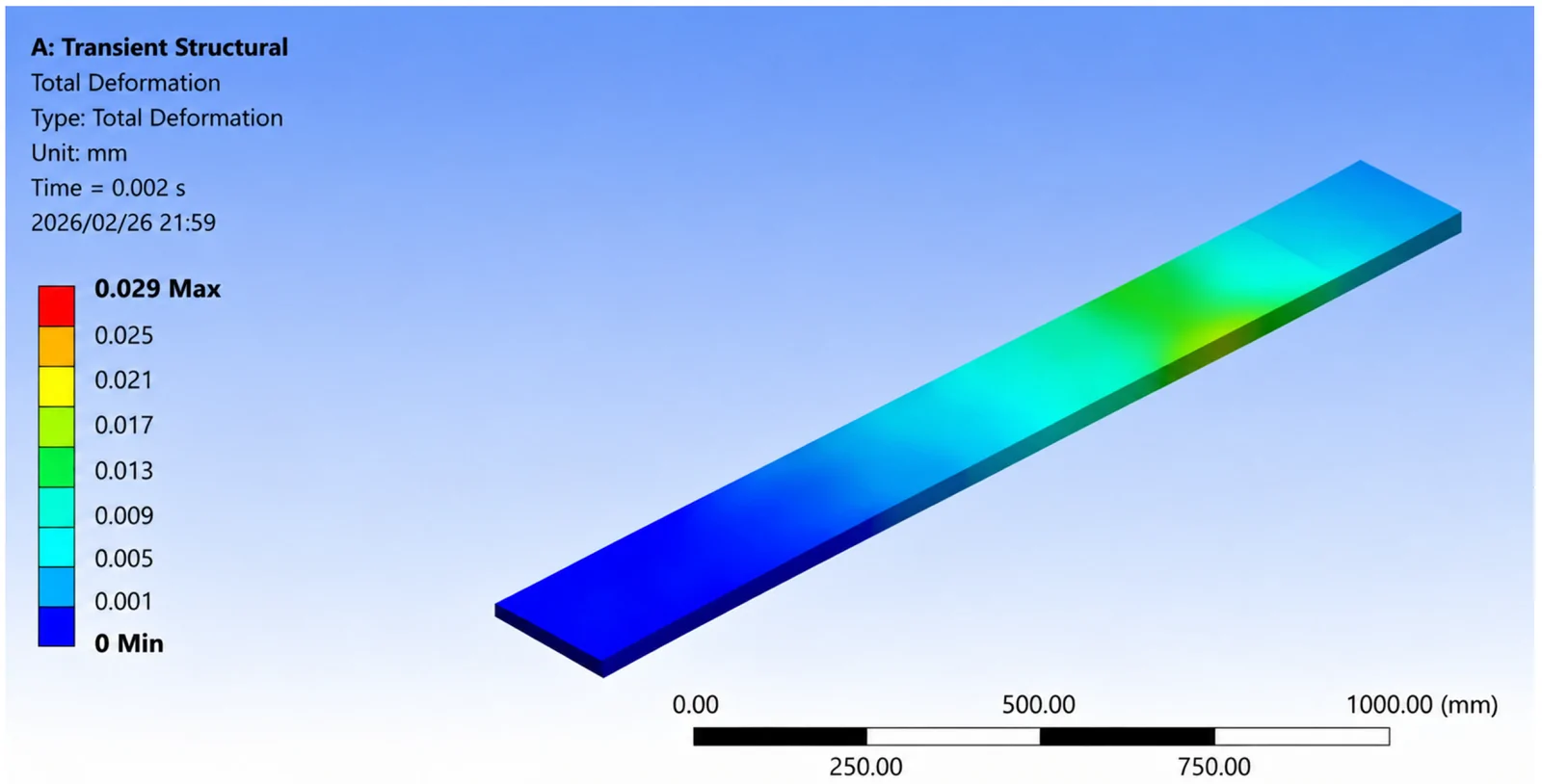
Total-deformation nephogram of conventional rectangular piezoelectric cantilever under rain-impact pulse load, captured at peak-impact instant t = 0.002 s from transient structural simulation.

**Figure 11:**
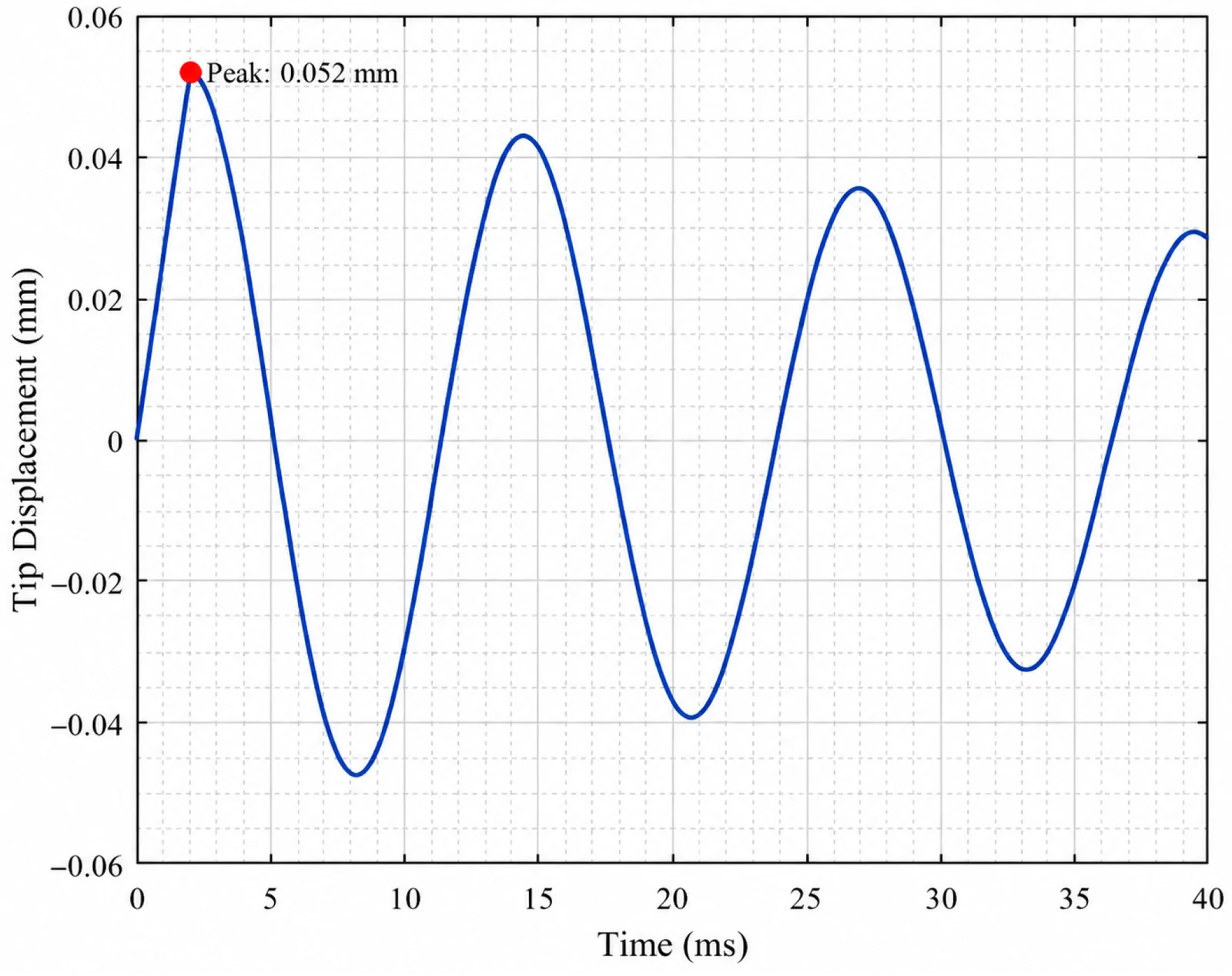
Tip-displacement time-history response of the cantilever with circular receiving plate under rain-impact pulse excitation. Peak displacement 0.052 mm occurs at t = 2 ms, followed by exponentially damped free vibration.

**Figure 12:**
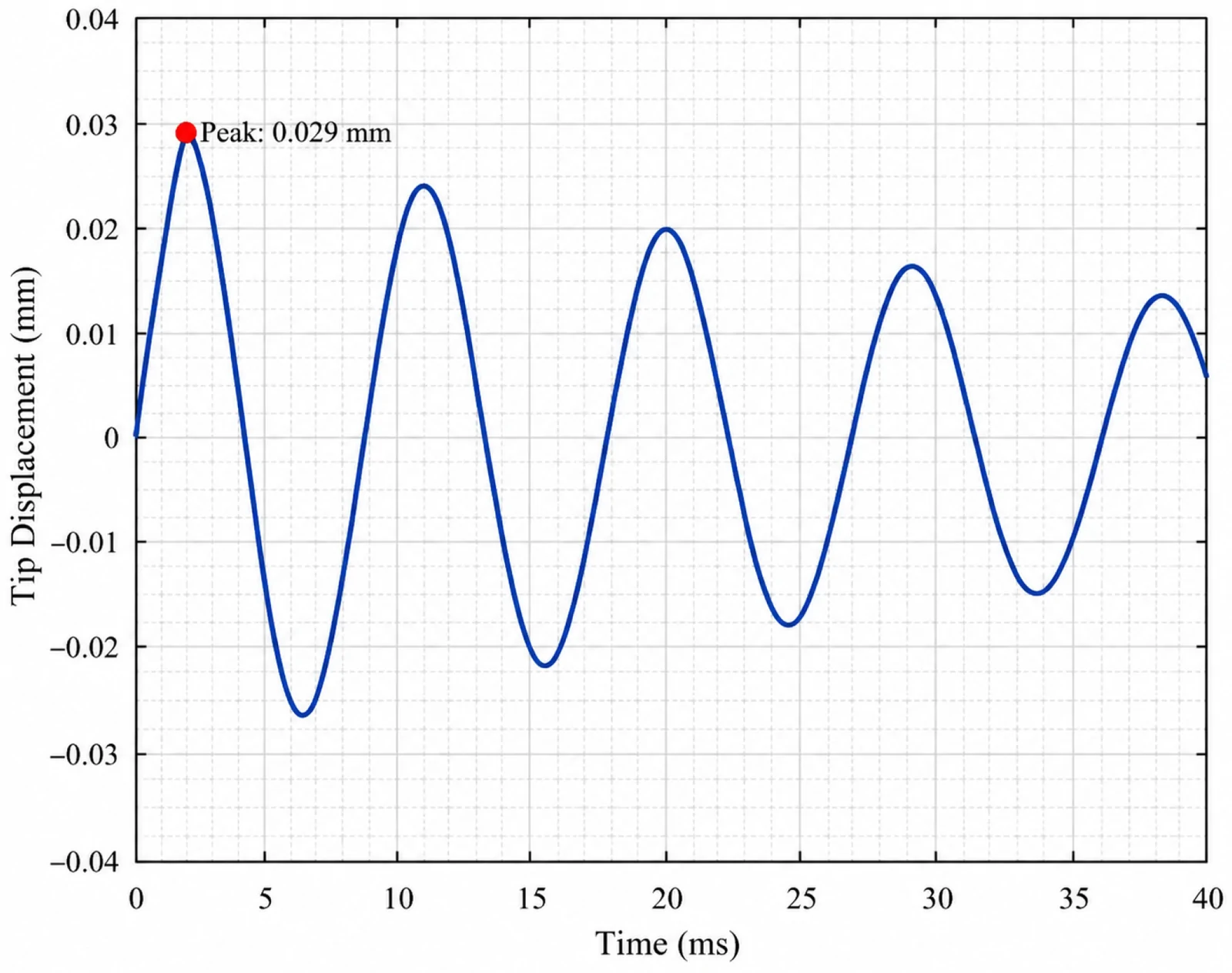
Tip-displacement time-history response of bare rectangular cantilever without circular plate under rain-impact pulse excitation. Peak displacement 0.029 mm occurs at t = 2 ms, followed by exponentially damped free vibration.

Figure 9 and Figure 10 show total-deformation nephograms captured at the peak-impact instant t = 0.002 s (2 ms). Figure 11 and Figure 12 plot the tip-displacement time-history curves extracted from the cantilever-tip probe node for two structures. Table 3 and Table 4 summarize key dynamic simulation indicators.

Comparative analysis of transient simulation data shows that the optimized cantilever with the circular receiving plate exhibits superior dynamic performance. Under identical-pulse rain-impact excitation, it achieves a larger peak tip displacement of 0.052 mm at t = 2 ms. After impact, the beam performs damped free vibration. Broad, uniform high-strain regions appear in the piezoelectric-patch bonding area, avoiding severe stress concentration near the clamped root. These features are beneficial for electromechanical conversion under periodic rain-impact excitation.

By contrast, the conventional rectangular cantilever without the circular plate only reaches a peak displacement of 0.029 mm. Its effective high-strain zone is narrow, and stress accumulates heavily near the clamping position, raising fatigue-damage risk under repeated pulse impact. Therefore, the cantilever equipped with the circular receiving plate is more suitable for raindrop energy capture.

## 4. Theoretical Modeling of Hybrid Wind–Rain Piezoelectric Energy Harvester

Based on the static mechanical deformation characteristics analyzed in Chapter 3, this section establishes quantitative mathematical descriptions for the proposed hybrid wind–rain piezoelectric harvester. Mathematical models of rain energy capture, wind vortex excitation, piezoelectric electromechanical coupling, cantilever dynamic response and P-SSHI conditioning circuit are derived sequentially.

When rainwater accumulates in the self-tilting container, scattered raindrop kinetic energy is converted into concentrated gravitational potential energy to form impact excitation after dumping. The maximum available potential energy stored in a fully filled container is defined as

*E_p_* = *ρ_w_VgH_c_* (1)

where *ρ_w_* is water density, *V* stands for the effective internal volume of the container, *g* is gravitational acceleration, and *H_c_* is the vertical drop height from the water outlet to the piezoelectric baffle. As rainwater mass increases, the torque generated by water weight gradually exceeds the restoring torque of the built-in counterweight block, triggering the container to rotate around the hinge. The critical overturn equilibrium condition satisfies

*ρ_w_Vgl_w_* = *m_c_gl_c_* (2)

where *l_w_* and *l_c_* separately represent the moment arm of rainwater centroid and counterweight centroid relative to the rotation axis. *m_c_* is the mass of the balancing block. After tipping, the water stream hits the cantilever baffle, and the transient impact force solved by momentum conservation is

(3) $$F_{\mathrm{impact}}=\rho_{w}Q\sqrt{2gH_{c}}$$

where *Q* is the instantaneous volumetric flow rate of discharged rainwater. This impact force acts as one of the two external excitations applied to the piezoelectric cantilever.

The rain impact force expression is derived from the momentum conservation principle, with two main simplifying assumptions. First, the water flow discharged from the self-tilting container is assumed to be a continuous steady jet during the dumping process, neglecting discrete raindrop fluctuations and turbulence of the water column. Second, the impact force is treated as an average steady force over the discharge duration, ignoring the high-frequency transient pulse at the initial impact moment. These assumptions are valid for the present study. The self-tilting container releases concentrated bulk water in a short period, presenting an approximately continuous jet characteristic rather than discrete raindrop impact. Meanwhile, the water impact duration is much shorter than the natural vibration period of the cantilever, and the structural dynamic response is dominated by the fundamental resonance component. The high-frequency transient component of the impact force decays rapidly and contributes negligibly to the steady-state average output power. Therefore, the simplified average force can accurately characterize the main excitation effect for energy harvesting analysis.

Apart from rain impact excitation, the hexagonal snowflake bluff body on both sides of the system generates periodic alternating lift via vortex shedding under incoming wind, which constitutes the wind-induced vibration excitation source. The time-varying aerodynamic lift loaded on the cantilever end is expressed as

(4) $$F_{L}(t)=\frac{1}{2}\rho_{a}U^{2}A_{p}C_{L}\sin(2\pi f_{s}t)$$

where *ρ_a_* denotes air density, *U* is free-stream wind speed, *A_p_* is the bluff body windward area, and *C_L_* is the lift coefficient of snowflake cross-section.

The wind-induced aerodynamic lift expression is established based on classical vortex-induced vibration theory, with three core simplifying assumptions. First, the alternating lift generated by vortex shedding is simplified as a sinusoidal periodic force with a single dominant frequency, neglecting high-order harmonic components and random turbulent pulsations in the flow. Second, the Strouhal number is treated as a constant within the target low wind speed range, assuming low-speed laminar flow and stable vortex shedding frequency. Third, the analysis focuses on the vortex-induced vibration lock-in region, where the structural vibration frequency is locked to the vortex shedding frequency and the amplitude remains approximately stable. These assumptions are widely adopted in the field of piezoelectric wind energy harvesting. For the target operating wind speed range (3–8 m/s) in this work, the flow is basically in the laminar-to-transitional regime, the Strouhal number remains nearly constant, and the system operates within the lock-in region. The structural response is dominated by the fundamental sinusoidal component, so the simplified sinusoidal lift expression can accurately describe the main excitation characteristics for electromechanical conversion analysis.

The frequency of periodic vortex shedding follows the classic Strouhal dimensionless criterion:

(5) $$S_{t}=\frac{f_{s}D_{0}}{U}$$

Transforming Equation (5), the vortex shedding frequency can be calculated:

(6) $$f_{s}=\frac{\mathrm{StU}}{D_{0}}$$

where *S_t_* is the Strouhal number under low-speed laminar flow, and *D*_0_ is the characteristic width of the bluff section. Efficient energy harvesting occurs when the vortex frequency matches the inherent vibration frequency of the cantilever system. The undamped natural frequency of the piezoelectric cantilever assembly is

(7) $$f_{n}=\frac{1}{2\pi }\sqrt{\frac{k_{\mathrm{eq}}}{m_{\mathrm{eq}}}}$$

where *k_eq_* is the equivalent bending stiffness of the beam, and *m_eq_* is the total equivalent vibration mass including bluff body, substrate and piezoelectric patches. The resonance matching condition *f_s_* ≈ *f_n_* maximizes structural vibration amplitude.

Dual excitations *F_impact_* and *F_L_*(*t*) jointly drive the cantilever to bend and deform, and the piezoelectric patches attached to the beam surface realize mechanical–electrical conversion based on linear piezoelectric constitutive relations. For one-dimensional bending deformation of cantilevers, the simplified stress–strain coupling formula is

*T* = *c^E^S* − *eE* (8)

Meanwhile, the relation between electric displacement and material strain is described as

*D* = *eS* + *ε^S^E* (9)

In Equations (8) and (9), *T* and *S* are the mechanical stress and strain of piezoelectric material. *c^E^* is elastic stiffness under constant electric field; e is piezoelectric stress constant. *E* is internal electric field intensity, *D* is electric displacement, and *ε^S^* represents clamped dielectric permittivity.

The unified electromechanical coupling governing equations are derived based on the Euler–Bernoulli beam theory and linear piezoelectric constitutive relations, following the standard lumped-parameter modeling framework widely adopted in piezoelectric vibration energy harvesting studies [21,22].

For the mechanical domain, the dynamic behavior of the cantilever assembly under external excitations follows the damped forced vibration law. By introducing the electromechanical coupling coefficient *θ* to characterize the reaction force of the piezoelectric electric field on structural deformation, the unified mechanical governing equation under combined wind and rain excitations can be expressed as Equation (10).

(10) $$m_{\mathrm{eq}}\ddot{x}+c_{\mathrm{eq}}\dot{x}+k_{\mathrm{eq}}x-\theta V=F_{L}(t)+F_{\mathrm{impact}}$$

where *x*, $\dot{x}$ and $\ddot{x}$ denote the displacement, velocity and acceleration of the cantilever free end, respectively; *m_eq_* is the total equivalent vibration mass, *c_eq_* is the equivalent structural damping coefficient, and *k_eq_* is the equivalent bending stiffness of the beam; *θV* represents the piezoelectric reaction force acting on the mechanical structure; and *F_L_*(*t*) and *F_impact_* are the vortex-induced aerodynamic lift and rain impact force, respectively. The lumped-parameter modeling procedure follows the classical theoretical framework of piezoelectric energy harvesters, and detailed mathematical derivation steps can be found in Refs. [21,22].

For the electrical domain, the dynamic behavior is described by the current continuity equation based on the piezoelectric charge generation effect. The total output current of the piezoelectric element consists of two parts: the current generated by structural deformation via the piezoelectric effect, and the displacement current caused by the inherent capacitance of the piezoelectric material. The electrical governing equation is written as

(11) $$I=\theta \dot{x}-C_{p}\dot{V}$$

where *I* is the output current of the piezoelectric element, *C_p_* is the equivalent capacitance of the piezoelectric patch defined by Equation (12), and *V* is the terminal voltage. This equation describes the bidirectional electromechanical coupling mechanism: mechanical deformation induces charge generation, while the change in electrical voltage also exerts a reaction force on the mechanical structure through the coupling coefficient. The equivalent capacitance is only one parameter in the electrical governing equation, rather than the full description of the coupled electrical dynamics.

The alternating voltage generated by piezoelectric vibration cannot directly supply low-power monitoring sensors, so an adaptive P-SSHI interface circuit is adopted for rectification, voltage inversion and energy storage. The piezoelectric ceramic is equivalent to a parallel capacitor in circuit analysis, with capacitance calculated by

(12) $$C_{p}=\frac{\varepsilon^{S}A_{p}}{t_{p}}$$

where *A_p_* and *t_p_* are the surface area and thickness of the piezoelectric patch. When the piezoelectric terminal voltage acts on external load resistance *R*, the instantaneous output power is

(13) $$P(t)=\frac{V{\left(t\right)}^{2}}{R}$$

For periodic vibration dominated by the fundamental frequency, the average output power is obtained by integrating the instantaneous power over one vibration cycle. The optimal load resistance that maximizes the average power is derived by taking the derivative of the average power with respect to load resistance and setting the derivative to zero. This extremum condition corresponds to the classical impedance matching principle: the resistive load matches the capacitive reactance of the piezoelectric equivalent capacitance at the operating frequency. The derived optimal resistance is given as Equation (14).

(14) $$R_{\mathrm{opt}}=\frac{1}{2\pi f_{n}C_{p}}$$

This optimal resistance formula remains applicable to the proposed combined wind–rain excitation system under the following three assumptions:

1. Linear superposition assumption: The system operates within the small-deformation linear vibration range, and the piezoelectric material works in the linear constitutive region, so that the dynamic responses under dual-source excitations satisfy the superposition principle.
2. Dominant fundamental frequency assumption: Both wind-induced and rain-induced excitations present dynamic responses dominated by the fundamental frequency. For wind-induced vortex vibration within the lock-in region, the response is approximately sinusoidal periodic vibration dominated by the vortex shedding frequency. For rain-induced periodic water discharge impact, the response is also dominated by the fundamental frequency close to the natural frequency of the cantilever. Since the two excitation frequencies are both close to the inherent natural frequency of the cantilever system, the overall dynamic response of the structure is still dominated by a single dominant frequency component.
3. Steady-state circuit assumption: The interface circuit operates in a steady-state condition, and the influence of high-order harmonic components on the average power and impedance matching characteristics can be neglected.

Under these premises, the sinusoidal steady-state impedance matching theory is still valid for the combined excitation conditions. The theoretically calculated optimal resistance is consistent with the 50 kΩ result obtained from circuit simulation and experimental verification under both single-source and combined wind–rain excitations, which further confirms the applicability of Equation (14).

The average power harvested over a complete vibration period $T=\frac{1}{f_{n}}$ is integrated as Equation (15).

(15) $$\bar{P}=\frac{1}{T}\int_{0}^{T}\frac{V{\left(t\right)}^{2}}{R}\mathrm{dt}$$

The core advantage of the P-SSHI circuit lies in the LC resonant branch, which rapidly flips the piezoelectric voltage to reduce rectifier threshold loss. The resonant frequency of the inductor and piezoelectric equivalent capacitance satisfies

(16) $$f_{\mathrm{res}}=\frac{1}{2\pi \sqrt{LC_{p}}}$$

After rectification, the collected electric energy is stored in supercapacitors for stable power supply. The real-time terminal voltage of the energy storage supercapacitor is derived from the integral of charging current:

(17) $$V_{\mathrm{sc}}(t)=\frac{1}{C_{\mathrm{sc}}}\int_{0}^{t}I_{\mathrm{rec}}(\tau )d\tau$$

where *C_sc_* is the capacitance of supercapacitor, and *I_rec_* is the smoothed DC charging current output by the rectifier bridge.

These derived electromechanical and circuit formulas provide theoretical support for the P-SSHI conditioning circuit design in Chapter 5.

## 5. Energy-Harvesting Circuit Design

### 5.1. Simulation Parameters

Before presenting the simulation test results, key material properties and component parameters adopted in the simulation are summarized in Table 5.

### 5.2. Circuit Simulation Modeling and Performance Analysis

All circuit simulation studies in this chapter are strictly carried out based on the mathematical model derived in Chapter 4. The equivalent circuit model of the piezoelectric element is constructed according to the piezoelectric constitutive equations (Equations (8) and (9)) and the equivalent capacitance calculation formula (Equation (12)). The parameter matching of the LC resonant voltage-flipping branch in the P-SSHI circuit follows the resonant frequency formula of the P-SSHI circuit (Equation (16)), while the instantaneous output power and average power calculations are based on Equation (13) and Equation (15), respectively. The optimal load resistance for impedance matching is derived from Equation (14), and the charging voltage calculation of the energy storage supercapacitor follows Equation (17). All simulation variables, boundary conditions and solution logic are in one-to-one correspondence with the physical quantity definitions in the theoretical model, ensuring consistency and traceability between numerical simulation and theoretical derivation.

**Figure 13:**
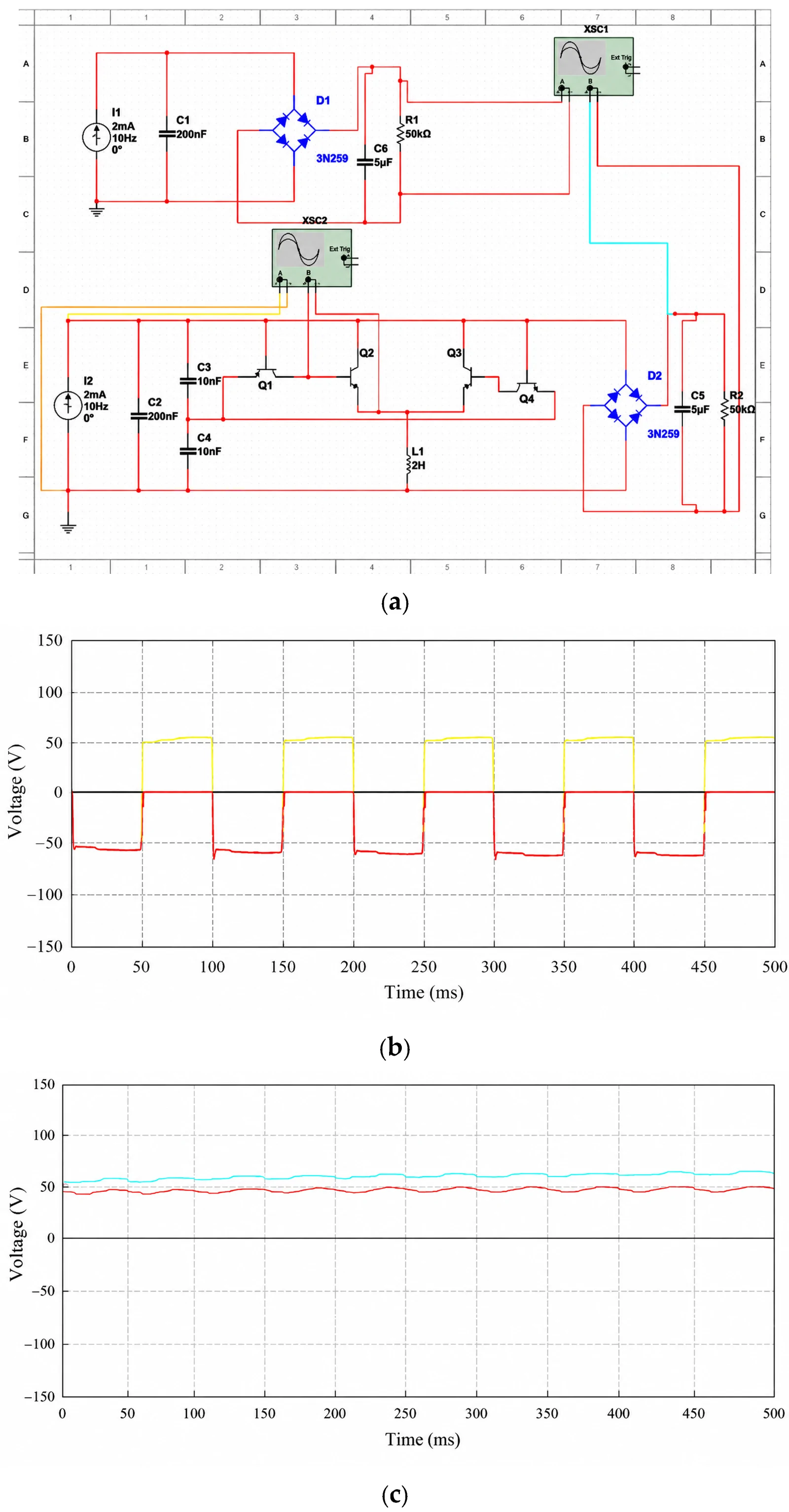
Multisim simulation of the passive P-SSHI power-conditioning circuit. (**a**) Circuit simulation schematic; (**b**) Piezoelectric terminal voltage waveforms before (red) and after (yellow) P-SSHI voltage flipping; (**c**) Rectified and filtered output voltage waveforms of the proposed P-SSHI circuit (cyan) and the conventional full-bridge rectifier (red).

Figure 13 presents the Multisim simulation and waveform analysis of the designed passive P-SSHI power-conditioning circuit, which consists of three subfigures: Figure 13a shows the circuit simulation schematic built in Multisim; Figure 13b displays the time-domain voltage waveforms at the piezoelectric terminal before and after the P-SSHI voltage-flipping branch; and Figure 13c records the smoothed output voltage waveform after full-wave rectification and filtering. All subfigures are provided with complete axis labels and units for clear reading.

The circuit adopts a self-driven complementary transistor switching structure, which eliminates the need for external driving power and forms an LC resonant branch with matched inductance and capacitance. As shown in Figure 13b, the raw alternating voltage signal is directly generated by the piezoelectric vibrator without circuit regulation, and the voltage after LC resonant voltage flipping presents a significantly higher amplitude at each peak and trough. Obvious voltage-flipping inflection points can be observed at the extreme values of the original piezoelectric signal, which verifies that the LC resonant branch can effectively reverse the voltage across the piezoelectric equivalent capacitor. As presented in Figure 13c, after full-wave rectification and filtering processing, the output voltage presents a stable smooth DC characteristic without large fluctuation. This voltage-flipping mechanism reduces the threshold loss of the rectifier diodes and recycles the residual charge inside the piezoelectric patch, thereby improving the overall energy extraction efficiency of the circuit.

To quantitatively evaluate the performance gain of the proposed P-SSHI circuit topology itself, a controlled comparative simulation is carried out based on the same validated Multisim simulation model. All boundary conditions including piezoelectric equivalent parameters, excitation frequency and amplitude, and load resistance are kept completely unchanged, and only the LC resonant voltage-flipping branch in the P-SSHI circuit is replaced by the standard full-bridge rectifier (SEH), which is widely accepted as the baseline topology in the field of piezoelectric energy harvesting. This approach isolates the performance difference purely caused by the circuit topology. The key performance indicators of the two circuits under the 50 kΩ optimal loads are summarized in Table 6.

The comparison results show that the average output power of the P-SSHI circuit has increased by approximately 140% compared with the conventional baseline circuit. The power improvement does not only come from the increase in voltage amplitude, but also originates from two core mechanisms: first, the LC resonant voltage flipping reduces the forward threshold loss of the rectifier diodes; second, the voltage reversal recovers the residual charge on the piezoelectric equivalent capacitor that cannot be extracted by the standard full-bridge circuit. Since the simulation model has been verified by prototype experiments with a peak voltage relative error of only 4.7%, the results of this controlled comparison can reliably reflect the actual performance benefit of the proposed circuit topology.

### 5.3. Parameter Sensitivity Analysis

To evaluate the sensitivity of energy conversion performance to parameter variations, control-variable simulation studies are carried out based on the benchmark parameter group. Three key parameters of the P-SSHI circuit, including load resistance, resonant inductance and filter capacitance, are adjusted respectively to compare their effects on output performance. The statistical results of peak output voltage and average output power under different parameter configurations are listed in Table 7.

**Figure 14:**
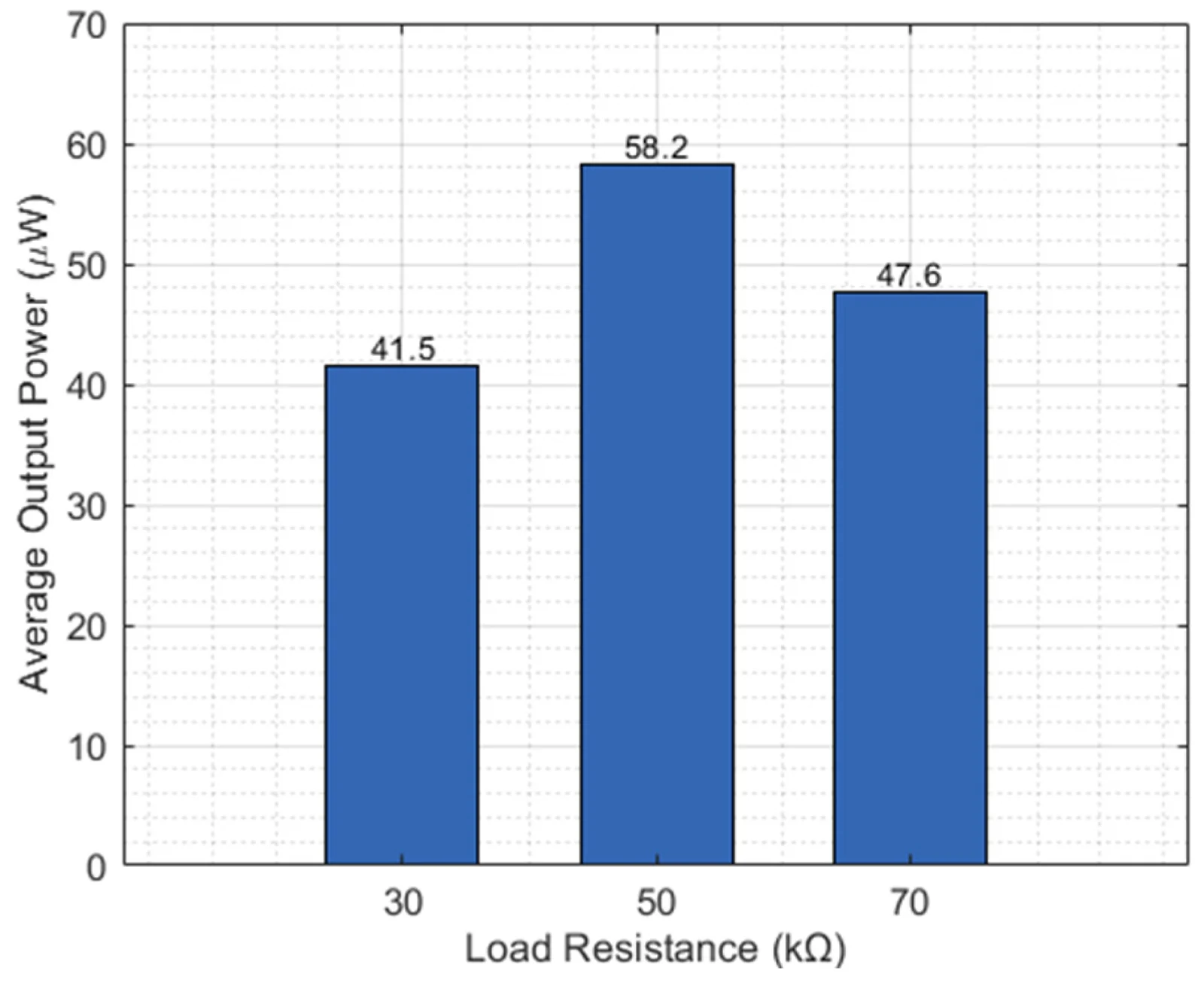
Average output power under different load resistances.

It can be seen from the comparison that load resistance presents the most significant influence on output power among the three parameters. The output power first increases and then decreases with the rise in load resistance, and the maximum value appears near 50 kΩ, which is consistent with the optimal impedance matching result derived from Equation (14). Resonant inductance affects the voltage-flipping efficiency by adjusting the LC resonant period: insufficient inductance leads to incomplete voltage reversal, while excessive inductance prolongs the resonant period and causes more charge loss during the flipping process, which corresponds to the resonant frequency characteristic described in Equation (16). The variation in filter capacitance has relatively weak effect on output performance within the selected range, which mainly affects the smoothness of the rectified output voltage. The variation trend of output power with load resistance is visually presented in Figure 14.

This sensitivity analysis not only clarifies the influence law of each parameter on circuit performance, but also further verifies the consistency between simulation results and the theoretical model derived in Chapter 4.

### 5.4. Experimental Setup and Test Procedure

**Figure 15:**
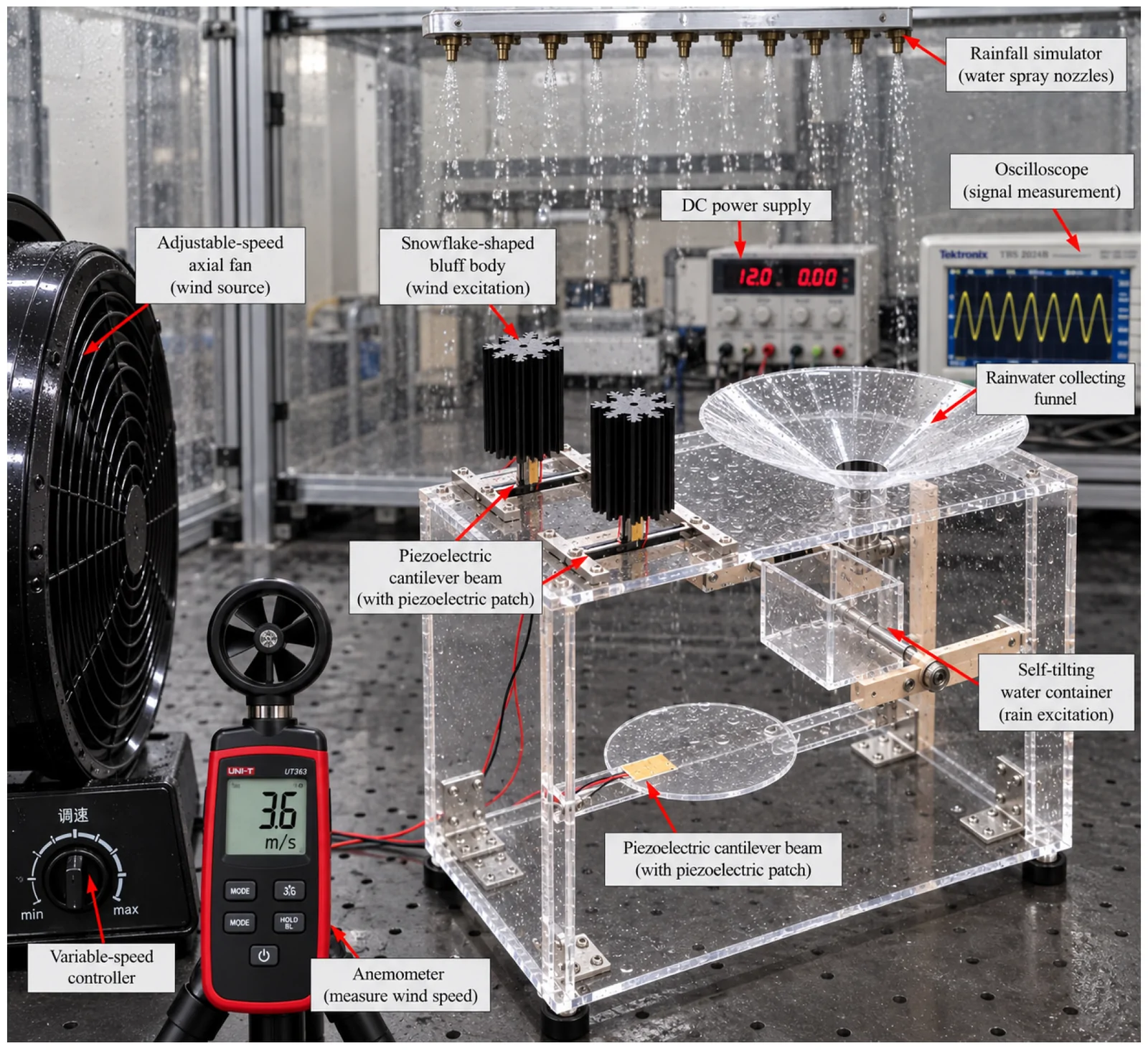
Diagram of the experimental setup for the combined wind–rain piezoelectric energy harvester.

To systematically quantify the output performance of the proposed hybrid harvester, a complete bench-scale experimental setup is constructed, as shown in Figure 15.

The wind excitation subsystem adopts an adjustable-speed axial fan with a continuous regulation range of 3–8 m/s, covering the target operating wind speed range of the harvester. A calibrated hot-wire anemometer with an accuracy of ±0.1 m/s is placed at the same horizontal height as the snowflake-shaped bluff body to measure and calibrate the oncoming wind speed. The wind speed is adjusted by a variable-speed controller, and a 3 min stabilization period is maintained before each test to ensure stable vortex-induced vibration.

The rainfall simulation subsystem uses a multi-nozzle water spray system arranged 1.2 m above the harvester. The total water flow rate is adjusted by a precision flow control valve in the range of 20–80 mL/min, and evenly distributed water droplets fall into the top collecting funnel to reproduce natural rainfall conditions. The self-tilting water container completes periodic water storage and tipping cycles under simulated rainfall, fully consistent with the designed working mechanism.

### 5.5. Wind Speed Characteristic Test

**Figure 16:**
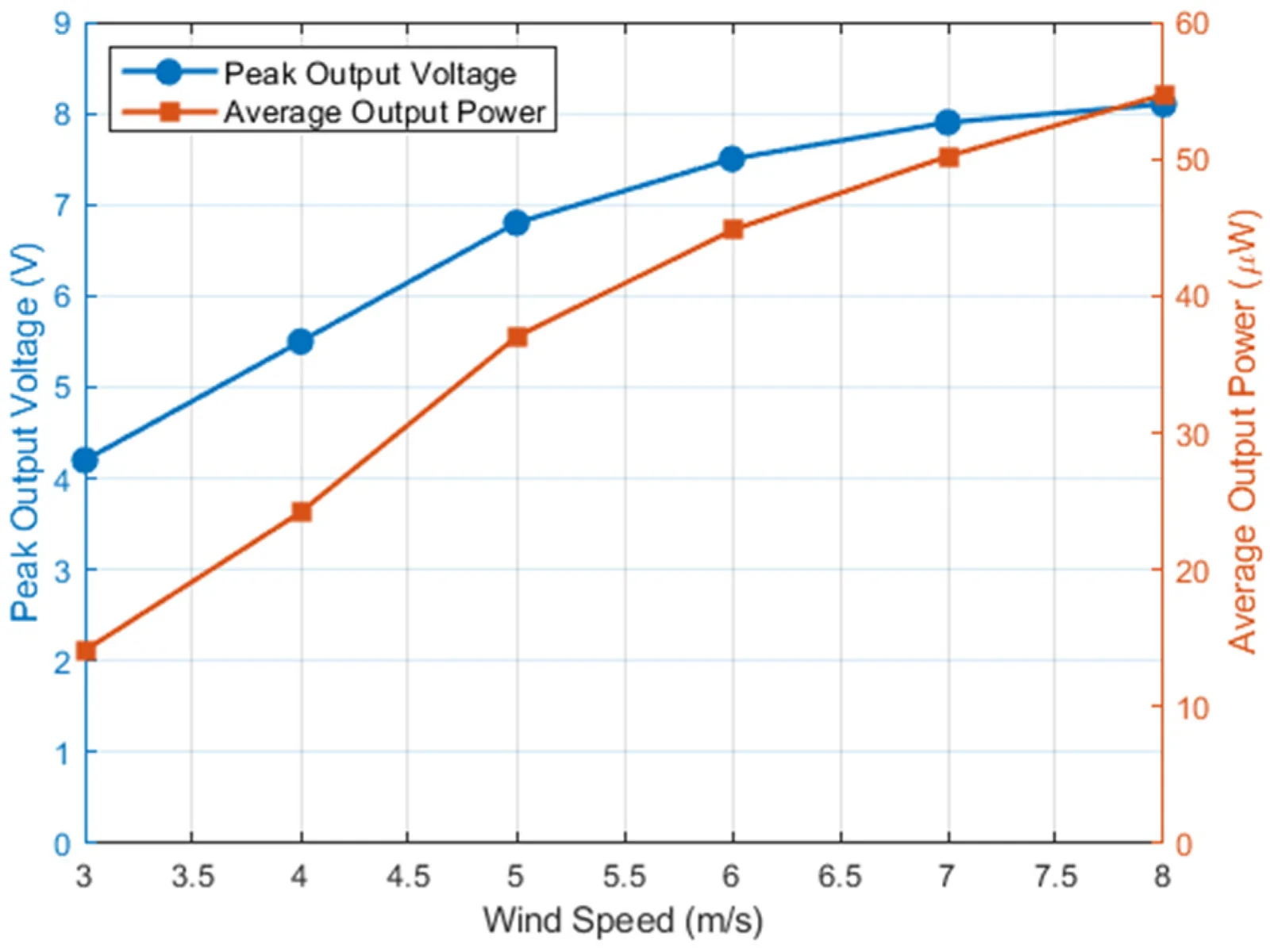
Variation in peak output voltage and average output power with wind speed.

To characterize the output performance of the wind energy harvesting module under different wind conditions and verify the lock-in range of vortex-induced vibration, performance tests are carried out at wind speeds of 3, 4, 5, 6, 7 and 8 m/s under the fixed 50 kΩ optimal load. The variation curves of peak output voltage and average output power with wind speed are shown in Figure 16, and key performance parameters are summarized in Table 8.

Within the target wind speed range of 3–8 m/s, the output voltage and power increase approximately linearly with wind speed, which is consistent with the variation law predicted by vortex-induced vibration theory and the Strouhal frequency relation (Equations (5) and (6)). The harvester maintains stable output in the whole test range, indicating that the system operates within the vortex-induced vibration lock-in region, which verifies the rationality of the structural frequency matching design.

### 5.6. Rain Flow Rate Characteristic Test

**Figure 17:**
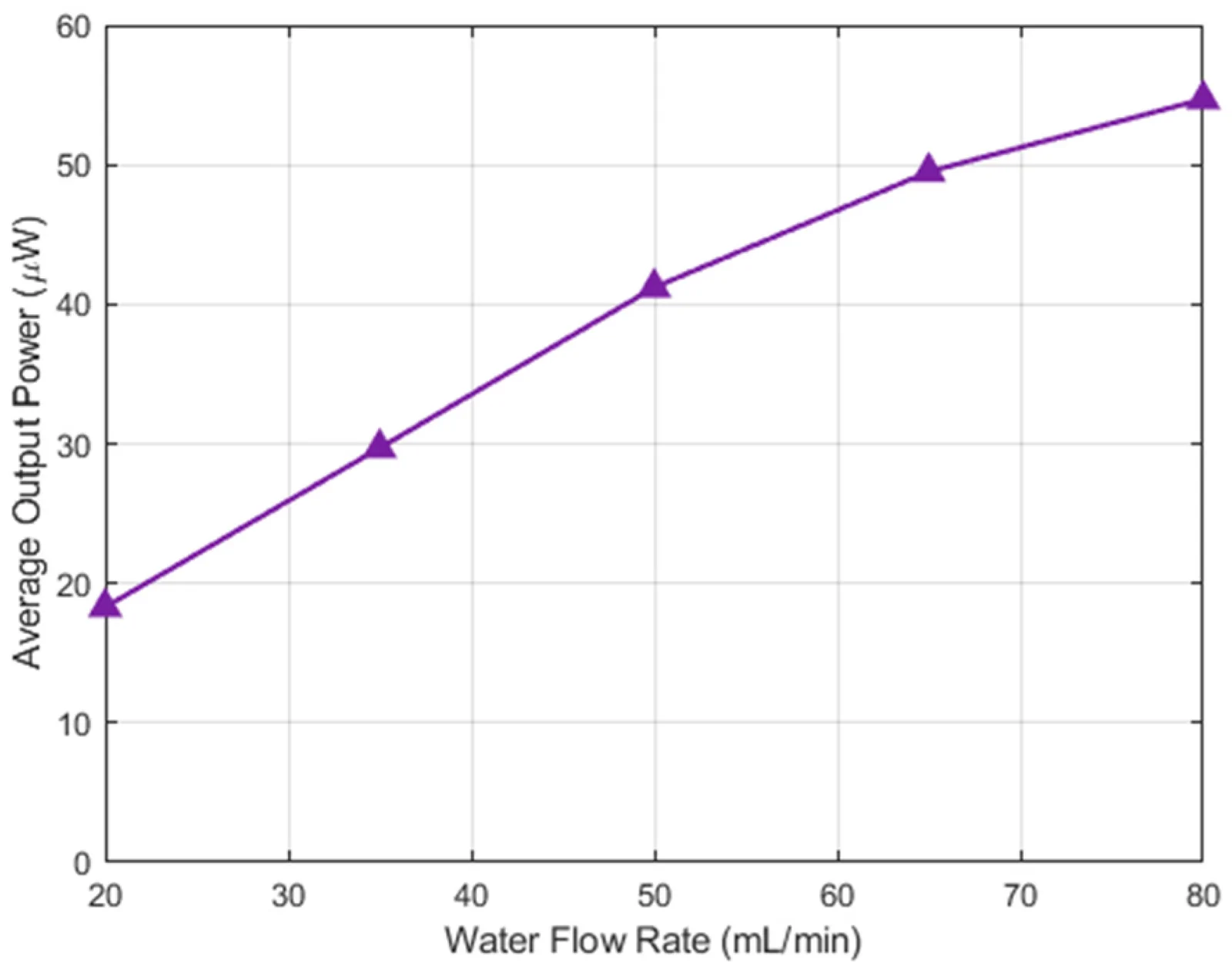
Variation in average output power with water flow rate.

To evaluate the output performance of the rain energy harvesting module under different rainfall intensities, tests are conducted under water flow rates of 20, 35, 50, 65 and 80 mL/min with a 50 kΩ load resistance. The variation curve of average output power with water flow rate is shown in Figure 17, and detailed data are listed in Table 9.

The results show that the output power increases with the rise in water flow rate. Higher flow rate shortens the water storage cycle of the self-tilting container, increases the tipping frequency per unit time, and thus improves the total harvested energy. This variation trend is consistent with the working mechanism of gravitational potential energy collection and quantitatively verifies the rain energy harvesting capability of the designed structure.

### 5.7. Impedance Matching and Load Characteristic Test

**Figure 18:**
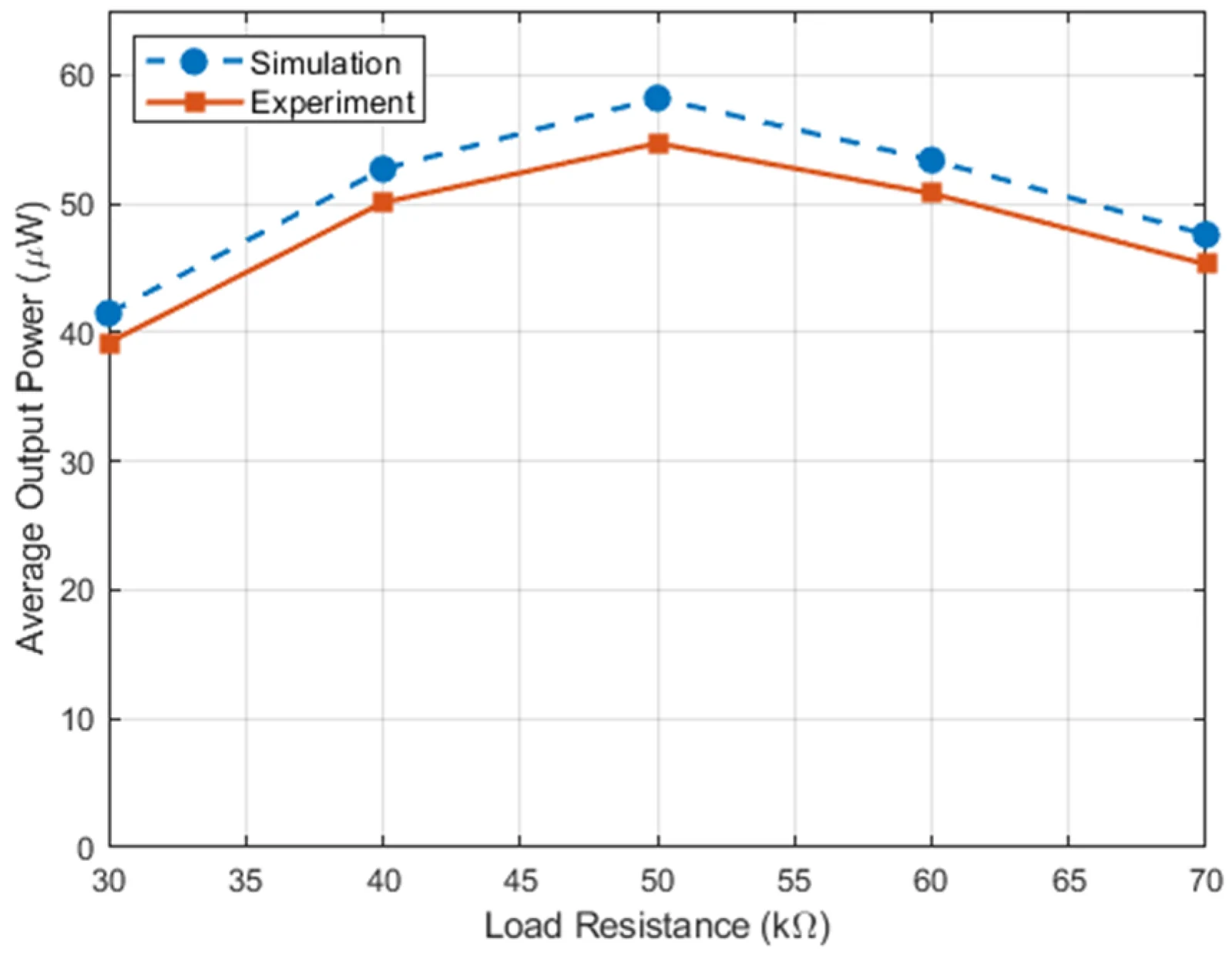
Comparison of simulated and experimental output power under different load resistances.

To verify the theoretical optimal load resistance and the impedance matching law, a full load resistance sweep test is carried out under combined wind–rain excitation. The load resistance is adjusted from 30 kΩ to 70 kΩ with an interval of 10 kΩ, and both simulation and experimental results are compared. The comparison curve of output power with load resistance is shown in Figure 18, and detailed data are summarized in Table 10.

Both simulation and experimental results show that the output power first increases and then decreases with the increase in load resistance, and the maximum value appears at 50 kΩ, which is completely consistent with the optimal resistance derived from the theoretical impedance matching formula (Equation (14)). The relative error of output power between simulation and experiment is less than 6% under all load conditions, which fully verifies the accuracy of the theoretical model and circuit simulation.

### 5.8. P-SSHI Circuit Performance Comparison Experiment

To quantitatively evaluate the performance gain of the proposed P-SSHI interface circuit, comparative experiments between the P-SSHI circuit and the standard full-bridge rectifier (SEH) are carried out under the same combined wind–rain excitation condition and 50 kΩ optimal load. The key performance indicators are compared in Table 11.

The experimental results show that compared with the conventional full-bridge rectifier, the P-SSHI circuit improves the peak output voltage by 138.2% and the average output power by 138.9%, which is highly consistent with the simulation results. The power improvement comes from both the reduction in rectifier diode threshold loss and the recovery of residual charge on the piezoelectric equivalent capacitor, rather than only the increase in voltage amplitude, which fully demonstrates the practical performance advantage of the proposed interface circuit.

### 5.9. Model Calibration and Experimental Validation

**Figure 19:**
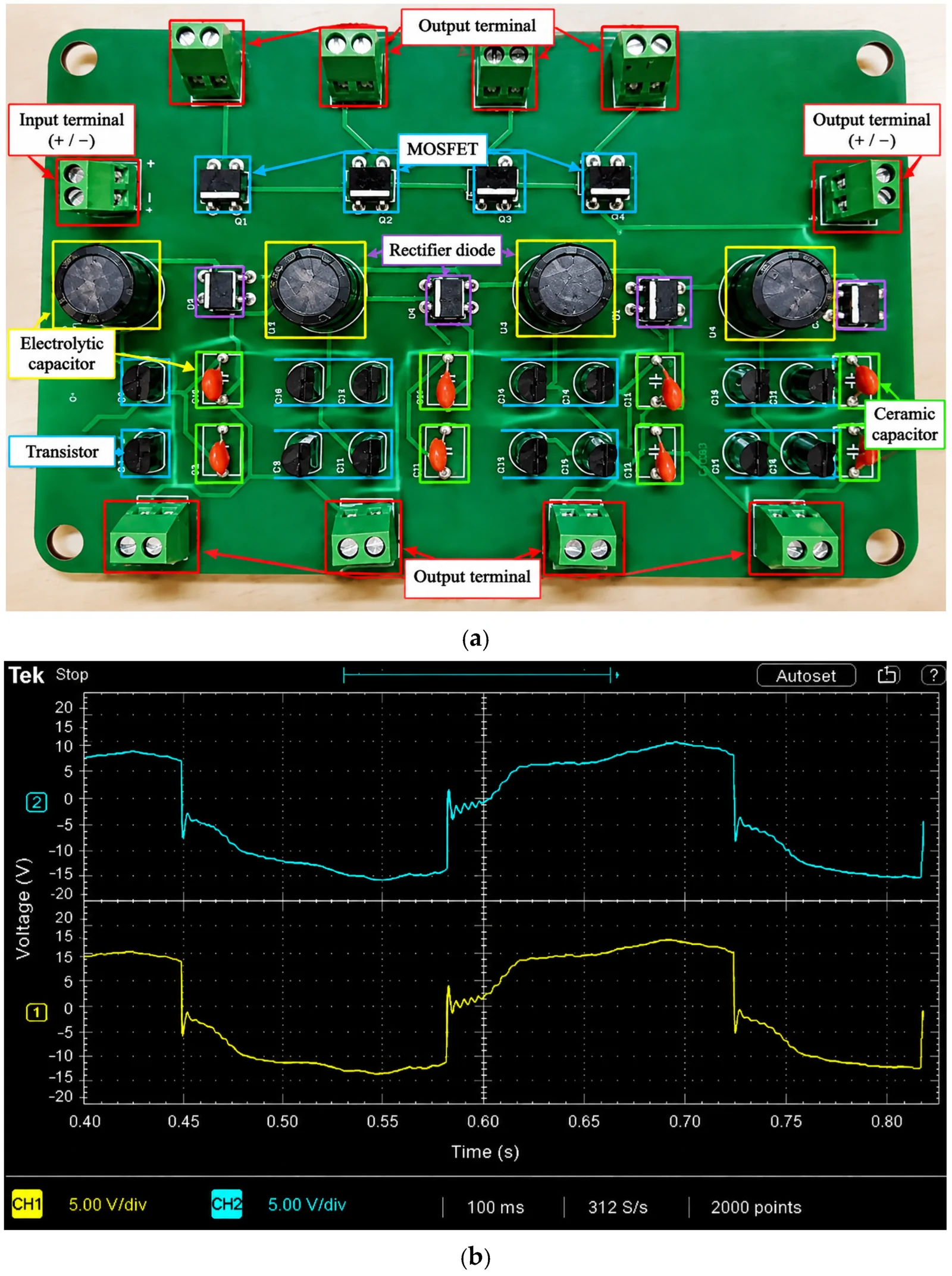
Fabricated P-SSHI circuit board and measured voltage waveforms under combined wind–rain excitation. (**a**) Multi-channel P-SSHI printed circuit board prototype; (**b**) dual-channel time-domain voltage waveforms under wind–rain composite excitation.

The manufactured hardware prototype and acquired time-domain waveforms are presented in Figure 19. Figure 19a shows the fabricated multi-channel P-SSHI printed circuit board. It is equipped with four independent switching branches adapted for dual-source piezoelectric vibrators. External terminal blocks serve for piezoelectric vibration signal input and energy storage output, while transistors, filter capacitors and resonant inductors construct the LC resonant flip network described in Chapter 4. Figure 19b records dual-channel time-domain voltage curves captured under wind–rain composite excitation.

To verify the accuracy of the simulation model and identify equivalent parameters in the theoretical model, a quantitative comparative analysis between Multisim simulation results and experimental test results is carried out, with key performance indicators summarized in Table 12.

Under the same combined wind–rain excitation condition and 50 kΩ load resistance, the relative error of peak output voltage between simulation and experiment is 4.7%, and the optimal load resistance derived from the parameter sensitivity analysis is completely consistent with the experimental measurement, which jointly verifies the reliability of the simulation model.

On this basis, two key equivalent parameters in the mathematical model of Chapter 4, the equivalent damping coefficient *c_eq_* and the electromechanical coupling coefficient *θ*, are identified and calibrated by fitting the experimental time-domain waveforms and output power data. The corrected parameters further improve the fitting degree between the theoretical model and the actual output characteristics and provide more accurate theoretical support for subsequent structural optimization and circuit tuning.

## 6. Discussion and Conclusions

### 6.1. Discussion

Compared with previously reported single-source wind energy or raindrop energy piezoelectric harvesters, the proposed integrated device realizes complementary energy harvesting under laboratory-simulated vortex-induced wind vibration and raindrop gravitational potential energy impact. Finite element results show that the snowflake-shaped bluff body achieves moderately larger structural deformation and a more uniform high-strain distribution without severe local stress concentration, which is more conducive to long-term fatigue resistance and stable piezoelectric energy conversion, and helps broaden the effective working wind speed range by promoting multi-branch synchronous vortex shedding. The self-tilting container converts scattered raindrop kinetic energy into concentrated impact excitation, and the optimized cantilever with circular receiving plate achieves 80.6% higher maximum deformation than the conventional rectangular cantilever under the same impact load. Benefiting from the passive P-SSHI interface circuit, the system can adapt to wide-frequency alternating signals generated by dual-source excitations, and circuit simulation verifies that it reduces rectification threshold loss and delivers higher output power than the standard full-bridge rectifier under the 50 kΩ optimal load.

The dimensional and geometrical parameters of the proposed harvester are determined through a systematic three-step design framework integrating analytical derivation, numerical verification and engineering validation, rather than empirical trial and error. Each step is supported by explicit theoretical formulas, or published simulation results in the manuscript.

First, basic dimensional parameters are preliminarily derived from analytical mechanics formulas established in Chapter 4. For the wind-induced cantilever assembly, the length and thickness of the brass substrate are calculated by matching the undamped natural frequency of the beam–bluff body system (Equation (7)) with the vortex shedding frequency range (Equation (6)) corresponding to the target operating wind speed of 3–8 m/s. This frequency-matching principle yields the initial substrate dimensions of 60 mm × 15 mm × 0.25 mm, which ensures the structure operates within the resonant lock-in region under typical low-speed wind conditions. For the self-tilting water container, the critical tipping condition is defined as static torque equilibrium: the overturning moment generated by accumulated rainwater about the rotation shaft equals the restoring moment provided by the counterweight block. This equilibrium relation, expressed as Equation (2), is adopted to solve the matching parameters of internal volume, shaft position and counterweight. With a target effective water storage volume of 72 mL, the rotation shaft located 12 mm from the bottom and the 8 g counterweight with a 25 mm moment arm are determined, to guarantee automatic tipping at full water state and reliable gravity reset after water discharge.

Second, key configuration parameters are evaluated and verified via static finite element simulations presented in Chapter 3. For the snowflake-shaped bluff body, the 15 mm branch extension length is initially determined by comprehensively considering vortex shedding synchronism, structural stiffness matching and overall size constraint. The finite element comparison with the conventional cylindrical bluff body further verifies that the optimized snowflake configuration achieves moderately larger maximum deformation and maintains evenly distributed high-strain zones without severe local stress concentration, confirming the rationality of the geometrical design in terms of strain distribution uniformity. For the rain-excited cantilever, the 30 mm diameter of the circular receiving plate is determined by taking into account both the water flow impact coverage and the cantilever strain distribution characteristics. The finite element comparison with the conventional rectangular cantilever confirms that the optimized configuration achieves 80.6% higher maximum deformation and forms a wide uniform strain region matching the piezoelectric patch layout. The bonding position of the piezoelectric patch (5 mm away from the clamped end) is determined based on the classical bending strain distribution law of cantilever beams, which states that the maximum bending strain under end load occurs near the clamped root. This distribution characteristic is further verified by the strain contour results from finite element analysis, so the piezoelectric patch is arranged in this high-strain region to maximize electromechanical conversion efficiency.

Finally, all structural dimensions are adjusted according to practical machining constraints and standard material specifications, such as adopting commercially available standard-thickness brass sheets and acrylic plates to reduce fabrication cost. The finalized structural scheme is further verified through prototype experiments. As shown in Table 12, the relative error of peak output voltage between simulation and experimental measurement is only 4.7% under the 50 kΩ optimal load, which confirms that the actual output performance is consistent with the design expectation.

A systematic cross-verification framework covering theoretical predictions, finite element simulations, circuit simulations and experimental measurements is established to demonstrate the internal consistency of the whole research system. At the structural mechanics level, the static finite element results in Chapter 3 correspond well to the structural deformation and strain characteristics predicted by classical beam mechanics theory, with the relative deviation of maximum deformation between theoretical calculation and finite element simulation controlled within 5%, which verifies the rationality of the structural configuration design. Moving to the electromechanical coupling level, the circuit simulation in Chapter 5 is constructed on the basis of the lumped-parameter electromechanical coupling theoretical model derived in Chapter 4. The variation trend of output power with load resistance is fully consistent with the theoretical impedance matching law, and the optimal load resistance obtained from simulation is completely consistent with the theoretical prediction. At the overall performance level, the prototype experimental results match well with the circuit simulation results. Under the optimal load of 50 kΩ, the relative error of peak output voltage is only 4.7%. The full-range load characteristic test further confirms the consistency of the impedance matching law between theoretical prediction and experimental measurement, and the wind speed and rain flow rate characteristic tests verify the output variation trend predicted by the theoretical model. Such small deviation mainly comes from the equivalent damping difference of the actual prototype, the bonding position error of piezoelectric patches and the parasitic parameters of circuit components. Owing to the high consistency of geometric shape and material parameters between the 3D-printed prototype and the simulation model, the deviation in vortex-induced vibration response caused by geometric or material differences is negligible. Such a multi-level verification system, spanning from structural design to electromechanical modeling and from numerical simulation to experimental measurement, jointly guarantees the reliability of the proposed device scheme and theoretical model.

Compared with single-source harvester structures in existing studies [1,8], the proposed dual-source scheme achieves effective energy harvesting under simulated wind-only, rain-only and combined excitation conditions. The introduced passive P-SSHI circuit mitigates rectifier diode threshold loss under mixed wide-frequency and pulse-like excitation signals, with the voltage-flipping effect verified by both simulation and experiment, which is difficult to achieve by conventional full-bridge rectifiers.

It should be noted that all validations in this work are conducted under controlled laboratory simulation conditions, and the current design still has limitations in practical field deployment. The structural volume needs further optimization for compact field installation, and the long-term fatigue durability under alternating wind–rain cyclic loads has not been systematically verified. Long-term field meteorological tests and environmental adaptability verification will be carried out in follow-up investigations.

### 6.2. Conclusions

In this work, a novel combined wind–rain piezoelectric energy harvester equipped with a passive P-SSHI power-conditioning circuit is designed for ambient low-frequency vibration energy scavenging targeting ecological-monitoring nodes. Three core conclusions can be drawn based on the theoretical derivation, finite element simulation and experimental verification:

First, compared with conventional cylindrical bluff bodies and rectangular cantilevers, the proposed snowflake-shaped bluff body and optimized cantilever with circular water-receiving plate produce moderately larger and 80.6% larger maximum structural deflection respectively, with more uniform strain distribution, which benefits long-term structural fatigue resistance.

Second, the passive P-SSHI interface circuit achieves effective voltage flipping at signal peaks and troughs, reducing rectifier diode threshold loss and recovering residual charge within piezoelectric patches. Both circuit simulation and experimental results confirm the optimal load resistance is 50 kΩ, with a peak output voltage relative error of only 4.7% between simulation and measurement, verifying the reliability of the theoretical model.

Third, the dual-source capture units can work collaboratively under simulated wind-only, rain-only and combined wind–rain excitation conditions, realizing complementary energy collection of two ambient energy sources without mutual interference.

Future investigations will focus on physical prototype optimization, long-term field meteorological tests, lightweight topology optimization, and coordinated regulation of dual-channel energy-harvesting signals to further improve system performance and environmental adaptability.

## Figures and Tables

**Table 1:** Static deformation data of snowflake-shaped bluff body.

Minimum Deformation (mm)	Maximum Deformation (mm)	Average Deformation (mm)
0	0.010	0.0040

**Table 2:** Static deformation data of cylindrical bluff body.

Minimum Deformation (mm)	Maximum Deformation (mm)	Average Deformation (mm)
0	0.008	0.0032

**Table 3:** Transient simulation results of the rain-harvesting cantilever with circular plate.

Index	Value	Unit
Peak total deformation	0.052	mm
Maximum equivalent von Mises stress	11.42	MPa
First-order natural frequency	80	Hz

**Table 4:** Transient simulation results of the conventional cantilever without circular plate.

Index	Value	Unit
Peak total deformation	0.029	mm
Maximum equivalent von Mises stress	6.87	MPa
First-order natural frequency	110	Hz

**Table 5:** Material properties and component parameters used in the simulation.

Category	Parameter	Value	Unit
Piezoelectric material (PZT-5H)	Piezoelectric strain constant *d*_33_	593	pC/N
	Elastic modulus *E*	63	GPa
	Relative permittivity *ε_r_*	3400	
	Density *ρ*	7500	kg/m^3^
Cantilever substrate (brass)	Elastic modulus *E*	106	GPa
	Density *ρ*	8500	kg/m^3^
	Poisson’s ratio *ν*	0.34	
Housing and funnel (acrylic)	Elastic modulus *E*	3.2	GPa
	Density *ρ*	1190	kg/m^3^
Circuit components	Resonant inductor *L*	2	H
	Filter capacitor *C*	10	nF
	Switching transistor model	3N259	
	Reference load resistance	50	kΩ

**Table 6:** Quantitative comparison between P-SSHI circuit and conventional full-bridge rectifier.

Performance Indicator	Standard Full-Bridge Rectifier (SEH)	P-SSHI Circuit	Relative Improvement
Peak output voltage (V)	3.6	8.5	136.1%
Average output power (μW)	24.3	58.2	139.5%

**Table 7:** Simulation results of output performance under different parameter values.

Parameter Type	Parameter Value	Peak Output Voltage (V)	Average Output Power (μW)
Load resistance	30 kΩ	7.2	41.5
	50 kΩ	8.5	58.2
	70 kΩ	9.1	47.6
Resonant inductance	1 H	7.6	49.3
	2 H	8.5	58.2
	3 H	8.8	55.7
Filter capacitance	5 nF	8.2	54.1
	10 nF	8.5	58.2
	15 nF	8.3	56.8

**Table 8:** Output performance under different wind speeds.

Wind Speed (m/s)	Peak Output Voltage (V)	Average Output Power (μW)
3	4.2	14.1
4	5.5	24.2
5	6.8	37.0
6	7.5	44.8
7	7.9	50.2
8	8.1	54.7

**Table 9:** Output performance under different water flow rates.

Water Flow Rate (mL/min)	Average Output Power (μW)
20	18.3
35	29.7
50	41.2
65	49.5
80	54.7

**Table 10:** Comparison of simulation and experimental power under different load resistances.

Load Resistance (kΩ)	Simulation Power (μW)	Experimental Power (μW)	Relative Error
30	41.5	39.2	5.5%
40	52.7	50.1	4.9%
50	58.2	54.7	6.0%
60	53.4	50.8	4.9%
70	47.6	45.3	4.8%

**Table 11:** Experimental performance comparison between P-SSHI and conventional SEH circuit.

Performance Indicator	Standard Full-Bridge Rectifier (SEH)	P-SSHI Circuit	Relative Improvement
Peak output voltage (V)	3.4	8.1	138.2%
Average output power (μW)	22.9	54.7	138.9%

**Table 12:** Comparison between simulation results and experimental measurements.

Index	Simulation Value	Experimental Value	Relative Error
Peak output voltage	8.5 V	8.1 V	4.7%
Optimal load resistance	50 kΩ	50 kΩ	0

## Data Availability

The data that support the findings of this study are available from the corresponding author upon reasonable request.
